# Conjugations of unitary operators, I

**DOI:** 10.1007/s13324-024-00924-z

**Published:** 2024-05-16

**Authors:** Javad Mashreghi, Marek Ptak, William T. Ross

**Affiliations:** 1 Département de mathématiques et de statistique, Université Laval, Québec, QC G1K 0A6 Canada; 2 Department of Applied Mathematics, University of Agriculture, ul. Balicka 253c, 30-198 Kraków, Poland; 3 Department of Mathematics and Computer Science, University of Richmond, Richmond, VA 23173 USA

**Keywords:** Complex symmetric operators, Unitary operators, 47B35, 47B02, 47A05

## Abstract

If *U* is a unitary operator on a separable complex Hilbert space $$\mathcal {H}$$, an application of the spectral theorem says there is a conjugation *C* on $$\mathcal {H}$$ (an antilinear, involutive, isometry on $$\mathcal {H}$$) for which $$ C U C = U^{*}.$$ In this paper, we fix a unitary operator *U* and describe *all* of the conjugations *C* which satisfy this property. As a consequence of our results, we show that a subspace is hyperinvariant for *U* if and only if it is invariant for any conjugation *C* for which $$CUC = U^{*}$$.

## Introduction

A version of the spectral theorem says that any unitary operator *U* on a separable complex Hilbert space $\mathcal {H}$ is unitarily equivalent to a multiplication operator $M_{\varphi } f = \varphi f$ on a Lebesgue space $L^2(X, \mu )$ [33, p. 13]. Here *X* is a compact Hausdorff space, $\mu$ is a finite positive Borel measure on *X*, and $\varphi \in L^{\infty }(X, \mu )$ is unimodular $\mu$-almost everywhere. If *J* is the mapping from $L^2(X, \mu )$ to itself defined by $(J f)(x) = \overline{f(x)}$, $x \in X$ (the bar denotes complex conjugation), then *J* is an antilinear, isometric, and involutive map and is called a *conjugation*. Furthermore, the conjugation *J* induces the adjoint identity $J M_{\varphi } J = M_{\overline{\varphi }} = M_{\varphi }^{*}$. Via unitary equivalence, given any unitary operator *U* on $\mathcal {H}$, this results in a conjugation *C* on $\mathcal {H}$ for which $C U C = U^{*}$ (see Lemma 2.3). In the parlance of operator theory, one says that *U* is a *C-symmetric operator* [18, 19]. As it turns out, there are many conjugations *C* on $\mathcal {H}$ for which $C U C = U^{*}$. The goal of this paper is to describe them all.

Towards this goal, let

$$\begin{aligned} \mathscr {C}_s(U):= \{C\hbox { is a conjugation on }\mathcal {H}: C U C = U^{*}\}. \end{aligned}$$

As discussed in the previous paragraph, $\mathscr {C}_s(U) \not = \varnothing$ (see also Proposition 2.5). On the other extreme, $\mathscr {C}_{s}(I)$, where *I* is the identity operator on $\mathcal {H}$, is simply the set of *all* conjugations on $\mathcal {H}$. The subscript *s*, for “symmetric”, in the above definition of $\mathscr {C}_{s}(U)$ might seem superfluous. However, in a follow up paper to this one [28], we will explore the set $\mathscr {C}_{c}(U)$ (notice the subscript *c*), the conjugations on $\mathcal {H}$ that commute with *U*, i.e., $C U C = U$. This paper provides several characterizations of $\mathscr {C}_s(U)$. One such characterization (Theorem 8.2) involves the spectral theorem. When one is fortunate enough to have a concrete spectral decomposition of a unitary operator, for example when *U* is an $n \times n$ unitary matrix, one can give a very tangible description of $\mathscr {C}_s(U)$. In particular, Theorem 4.2 says that if *U* is an $n \times n$ unitary matrix with spectral decomposition

$$\begin{aligned} U = W \begin{bmatrix} \xi _1 I_{n_1} &{} &{} &{} \\ &{} \xi _2 I_{n_2} &{} &{} \\ &{} &{} \ddots &{} \\ &{} &{} &{} \xi _d I_{n_d} \end{bmatrix} W^{*}, \end{aligned}$$

where *W* is an $n \times n$ unitary matrix, $\xi _1, \ldots , \xi _d$ ($1 \leqslant d \leqslant n$) are the distinct eigenvalues of *U*, $I_{n_j}$ is the $n_j \times n_j$ identity matrix, and $n_1 + n_2 + \cdots + n_d = n$, then any conjugation *C* on $\mathbb {C}^n$ for which $C U C = U^{*}$ must take the form

1.1 $$\begin{aligned} C = W \begin{bmatrix} V_1 &{} &{} &{} \\ &{} V_2 &{} &{} \\ &{} &{} \ddots &{}\\ &{} &{} &{} V_d \end{bmatrix} J W^{*}, \end{aligned}$$

where each $V_j$ is an $n_j \times n_j$ unitary matrix satisfying $V_{j}^{t} = V_{j}$ (*t* denotes the transpose of a matrix), and *J* denotes the conjugation on $\mathbb {C}^n$ defined by

$$\begin{aligned} J [x_1 \; x_2 \cdots x_n]^{t} = [\overline{x_1} \; \overline{x_2} \cdots \overline{x_n}]^{t}. \end{aligned}$$

A version of (1.1) can be obtained in the infinite dimensional setting when the eigenvectors for *U* are complete in $\mathcal {H}$. In particular, we give a concrete description of $\mathscr {C}_s(U)$ when *U* is the Fourier–Plancherel transform on $L^2(\mathbb {R})$ (Example 4.4) and when *U* is the Hilbert transform on $L^2(\mathbb {R})$ (Example 4.6).

The main driver of nearly all the results in this paper is a measure-theoretic decomposition of any $C \in \mathscr {C}_s(U)$ given in Theorem 3.2. This enables us to examine manageable pieces of *C* of $\mathcal {H}$ on certain reducing subspaces of *U*.

Another description of $\mathscr {C}_s(U)$ relies less on the spectral decomposition of a unitary operator *U*, which is often quite intangible in the general setting, and more on some special properties of *U*. For example, if *U* is a *bilateral shift* (see Definition 6.1), $\mathscr {C}_s(U)$ was described in [5] (see also Theorem 6.4). In particular, when *U* is *the* bilateral shift $(M_{\xi } f)(\xi ) = \xi f(\xi )$ on $L^2(m, \mathbb {T})$ (*m* is normalized Lebesgue measure on the unit circle $\mathbb {T}$), then $C \in \mathscr {C}_s(U)$ if and only if $C = M_{u} J$, where *J* is the conjugation on $L^2(m, \mathbb {T})$ given by $J f = \overline{f}$ and $u \in L^{\infty }(m, \mathbb {T})$ is unimodular almost everywhere on $\mathbb {T}$ (see Example 6.6).

The unitary multiplication operator $M_{\psi }$ on $L^2(m, \mathbb {T})$, where $\psi$ is an inner function (a bounded analytic function on $\mathbb {D}$ whose radial boundary values on $\mathbb {T}$ have modulus one *m*-almost everywhere [10, 23]), turns out to be a bilateral shift [29, Proposition 5.17] and a concrete description of $\mathscr {C}_s(M_{\xi })$ is given in Theorem 7.8. In fact (see Remark 7.1), such $M_{\psi }$ serve as models for all bilateral shifts and so, in a way, Theorem 7.8, is a canonical model for $\mathscr {C}_s(U)$ for any bilateral shift *U*.

As a byproduct of several results in this paper, we can connect conjugations with the hyperinvariant subspaces of a unitary operator *U* on $\mathcal {H}$ (those subspace which are invariant for any bounded operator on $\mathcal {H}$ commuting with *U*). Certainly the invariant and hyperinvariant subspaces of unitary operators have been discussed before (for example [8, 9, 31]). Here we connect them with conjugations in the following way. For a (closed) subspace $\mathcal {M}$ of $\mathcal {H}$, the following are equivalent: (i) $\mathcal {M}$ is hyperinvariant for *U*; (ii) $C \mathcal {M} \subseteq \mathcal {M}$ for every $C \in \mathscr {C}_{s}(U)$. This result is obtained, along with several other equivalent conditions covered in this paper, via Theorems 3.2, 3.8, and 8.6 and is officially stated in Theorem 8.8.

## Basic facts about conjugations and spectral measures

*All Hilbert spaces* $\mathcal {H}$ *in this paper will be complex and separable.* Let $\mathcal {B}(\mathcal {H})$ denote the set of all bounded linear transformations on $\mathcal {H}$ and $\mathscr {A}\!\mathcal {B}(\mathcal {H})$ denote the set of all *bounded antilinear transformations* on $\mathcal {H}$. By this we mean that $C \in \mathscr {A}\!\mathcal {B}(\mathcal {H})$ when $C(\textbf{x} + \alpha \textbf{y}) = C \textbf{x} + \overline{\alpha } C \textbf{y}$ for all $\textbf{x}, \textbf{y} \in \mathcal {H}$ and $\alpha \in \mathbb {C}$ (*C* is antilinear) and $\sup \{\Vert C \textbf{x}\Vert : \Vert \textbf{x}\Vert = 1\}$ is finite (*C* is bounded). We say that $C \in \mathscr {A}\!\mathcal {B}(\mathcal {H})$ is a *conjugation* if *C* satisfies the two additional conditions $\Vert C \textbf{x}\Vert = \Vert \textbf{x}\Vert$ for all $\textbf{x} \in \mathcal {H}$ (*C* is isometric) and $C^2 = I$ (*C* is involutive). By the polarization identity, a conjugation also satisfies

2.1 $$\begin{aligned} \langle C \textbf{x}, C \textbf{y}\rangle = \langle \textbf{y}, \textbf{x}\rangle \; \text{ for } \text{ all }\,\textbf{x}, \textbf{y} \in \mathcal {H}. \end{aligned}$$

Conjugations play an important role in operator theory and were initially studied in [13, 14, 17–19]. More recently, conjugations were explored in various settings and applications in [4, 5, 11, 12, 27, 32].

### Example 2.2

Many types of conjugations were outlined in [17–19]. Below are a few basic ones that are relevant to this paper.

- As discussed in the introduction, the mapping $C f = \bar{f}$ defines a conjugation on $L^2(X, \mu )$. In particular, the mapping $C [x_1 \; x_2 \; \cdots \; x_n]^{t} = [\overline{x_1} \; \overline{x_2} \; \cdots \; \overline{x_n}]^t$ defines a conjugation on $\mathbb {C}^n$. Throughout this paper we will use the notation *t* to represent the transpose of a matrix. In addition, vectors in $\mathbb {C}^n$ will be viewed as column vectors since, for an $n \times n$ matrix *A* of complex numbers, we will often consider the linear transformations on $\mathbb {C}^n$ defined by $\textbf{x} \mapsto A \textbf{x}$.
- One can consider the conjugations $(C f)(t) = \overline{f(t)}$ and $(C f)(t) = \overline{f(-t)}$ on $L^2(\mathbb {R})$. These were used in [1, 2] to study symmetric operators and their connections to physics.
- If *u* is an inner function, $H^2$ is the standard Hardy space, and $\mathcal {K}_{u}:= H^2 \ominus uH^2$ is the model space associated with *u* (considering everything as a subspace of $L^2(m, \mathbb {T})$ via radial boundary values), then $(C f)(\xi ) = u(\xi ) \overline{\xi f(\xi )}$ defines a conjugation on $\mathcal {K}_u$ [15, Ch. 8].

This next lemma enables us to transfer a conjugation on one Hilbert space to a conjugation on another. The (easy) proof is left to the reader.

### Lemma 2.3

Suppose $\mathcal {H}$ and $\mathcal {K}$ are Hilbert spaces and $V: \mathcal {H} \rightarrow \mathcal {K}$ is a unitary operator. If *C* is a conjugation on $\mathcal {H}$ then $VCV^{*}$ is a conjugation on $\mathcal {K}$.

For a conjugation *C* on $\mathcal {H}$ and an $A \in \mathcal {B}(\mathcal {H})$, we say that *A* is *C*-*symmetric* if $C A C = A^{*}$. A multitude of operators from a variety of settings enjoy this property [17, 18, 21, 22]. Here are a few examples that are relevant to this paper.

### Example 2.4

- If $\varphi \in L^{\infty }(X, \mu )$ and $M_{\varphi }$ denotes the multiplication operator $M_{\varphi } f = \varphi f$ on $L^2(X, \mu )$, then the conjugation *C* from Example 2.2(a) satisfies $C M_{\varphi } C = M_{\overline{\varphi }} = M_{\varphi }^{*}.$ Thus, $M_{\varphi }$ is *C*-symmetric. For a normal operator $N \in \mathcal {B}(\mathcal {H})$, the spectral theorem says that *N* is unitarily equivalent to $M_{\varphi }$ on $L^2(X, \mu )$ for some finite positive Borel measure on some compact Hausdorff space *X*. Now use the discussion above and Lemma 2.3 to see that *N* is a *C*-symmetric operator for some conjugation *C* on $\mathcal {H}$.
- The conjugation $(Cf)(x) = \overline{f(-x)}$ from Example 2.2(b) makes the translation operator $(U f)(x) = f(x - 1)$ on $L^2(\mathbb {R})$ a *C*-symmetric unitary operator. This conjugation also makes the Hilbert transform a *C*-symmetric unitary operator.
- The conjugation $(C f)(t) = \overline{f(t)}$ from Example 2.2(b) makes the Fourier–Plancherel transform a *C*-symmetric unitary operator on $L^2(\mathbb {R})$.

Though not used in this paper, but certainly in our follow up paper [28], we recall the following result from [24] which shows that any unitary operator can be built from conjugations. We include a short proof (slightly different from the original) for the reader’s convenience.

### Proposition 2.5

For each unitary operator *U* on $\mathcal {H}$, there are conjugations $J_1$ and $J_2$ on $\mathcal {H}$ such that $U=J_1J_2$. Moreover, $J_1, J_2 \in \mathscr {C}_{s}(U)$.

### Proof

For a given unitary *U* on $\mathcal {H}$, we can use the spectral theorem argument from Example 2.4(a) (and also from the introduction) to produce a conjugation $J_1$ on $\mathcal {H}$ such that $J_1 U J_1 = U^{*}$. Now observe that $J_2:= U^{*} J_1$ is a conjugation, $J_2 U J_2 = U^{*}$, and $J_1 J_2 = U$.

The following result will be used from time to time in this paper.

### Proposition 2.6

Suppose *U*, *V*, *W* are unitary operators on $\mathcal {H}$ such that $W U W^{*} = V$. Then $W \mathscr {C}_{s}(U) W^{*} = \mathscr {C}_{s}(V)$.

The following result from [20, Lemma 3.2], which will be generalized below, gives an explicit description of all the conjugations on $\mathbb {C}^n$.

### Proposition 2.7

A mapping *C* on $\mathbb {C}^n$ is a conjugation if and only if $C = V J$, where *V* is an $n \times n$ unitary matrix with $V^{t} = V$ and *J* is the conjugation on $\mathbb {C}^n$ defined by $J[x_1\; x_2\; \cdots \; x_n]^{t} = [\overline{x_1}\; \overline{x_2}\; \cdots \; \overline{x_n}]^{t}$, i.e.,

$$\begin{aligned} C[x_1 \; x_2 \; \cdots x_n]^{t} = V [\overline{x_1} \; \overline{x_2} \; \cdots \overline{x_n}]^{t}. \end{aligned}$$

Our discussion below needs a generalization of the previous result for $\mathbb {C}^n$ to an abstract (separable) Hilbert space $\mathcal {H}$, but, of course, we need a substitute for $V^{t}$ since the “transpose” of a linear operator *V* on $\mathcal {H}$ needs a proper definition (as opposed to the adjoint $V^{*}$ which has a clear and well understood definition). For a fixed orthonormal basis $\mathscr {B} = \{\textbf{u}_j\}_{j \geqslant 1}$ for $\mathcal {H}$ and an $A \in \mathcal {B}(\mathcal {H})$, we use the notation

$$\begin{aligned}{}[A]_{\mathscr {B}} = [a_{mn}]_{m, n \geqslant 1} = [\langle A \textbf{u}_{n}, \textbf{u}_{m}\rangle ]_{m, n \geqslant 1} \end{aligned}$$

to denote the matrix representation of *A* with respect to $\mathscr {B}$, considered as a bounded operator on the sequence space

2.8 $$\begin{aligned} \ell ^{2}_{+}:= \left\{ \textbf{x} = [x_1 \; x_2 \; \cdots ]^{t}, x_j \in \mathbb {C}: \Vert \textbf{x}\Vert _{\ell ^{2}_{+}} = \left( \sum _{j \geqslant 1} |x_j|^2\right) ^{1/2} < \infty \right\} \end{aligned}$$

by $\textbf{x} \mapsto [A]_{\mathscr {B}} \textbf{x}$. For this fixed orthonormal basis $\mathscr {B}$, we also define a conjugation $J_{\mathscr {B}}$ on $\mathcal {H}$ by

2.9 $$\begin{aligned} J_{\mathscr {B}}\left( \sum _{j \geqslant 1} c_j \textbf{u}_{j}\right) = \sum _{j \geqslant 1} \overline{c_j} \textbf{u}_j. \end{aligned}$$

In other words, $J_{\mathscr {B}}$ fixes every basis element $\textbf{u}_j$ and extends antilinearly to $\mathcal {H}$. Here is a version of Proposition 2.7 for a general, possibly infinite dimensional, separable Hilbert space.

### Proposition 2.10

For an orthonormal basis $\mathscr {B} = \{\textbf{u}_j\}_{j \geqslant 1}$ for a separable Hilbert space $\mathcal {H}$, the mapping *C* is a conjugation on $\mathcal {H}$ if and only if there is a unitary operator *V* on $\mathcal {H}$ such that $[V]_{\mathscr {B}}^{t} = [V]_{\mathscr {B}}$ and $C = V\!J_{\mathscr {B}}$.

### Proof

Suppose *C* is a conjugation on $\mathcal {H}$. Then $V = C\!J_{\mathscr {B}}$ is a unitary operator on $\mathcal {H}$ (since it is linear, isometric, and onto). Moreover, applying (2.9) and then (2.1), we see that

$$\begin{aligned} \langle V \textbf{u}_{m}, \textbf{u}_{n}\rangle&= \langle C\!J_{\mathscr {B}} \textbf{u}_{m}, \textbf{u}_{n}\rangle \\&= \langle C \textbf{u}_{m}, \textbf{u}_{n}\rangle \\&= \langle C \textbf{u}_{n}, \textbf{u}_{m}\rangle \\&= \langle C\!J_{\mathscr {B}} \textbf{u}_{n}, \textbf{u}_{m}\rangle \\&= \langle V \textbf{u}_{n}, \textbf{u}_{m}\rangle . \end{aligned}$$

Thus $$[V]_{\mathscr {B}}^{t} = [V]_{\mathscr {B}}$$ and $$C = V\!J_{\mathscr {B}}$$.

Conversely, if *V* is a unitary operator on $\mathcal {H}$ with $[V]_{\mathscr {B}}^{t} = [V]_{\mathscr {B}}$, define $C = V\!J_{\mathscr {B}}$. Observe that *C* is antilinear and isometric. We just need to verify that $C^2 = I$. Since *C* is antilinear, note that $C^2 = V\!J_{\mathscr {B}} V\!J_{\mathscr {B}}$ is a bounded *linear* operator on $\mathcal {H}$. Moreover, since $J_{\mathscr {B}} V\!J_{\mathscr {B}}$ is also a bounded linear operator on $\mathcal {H}$, we see that

2.11 $$\begin{aligned}{}[C^2]_{\mathscr {B}} = [V]_{\mathscr {B}} [J_{\mathscr {B}} V\!J_{\mathscr {B}}]_{\mathscr {B}}. \end{aligned}$$

Now observe that for all $m, n \geqslant 1$, again making use of (2.1) and (2.9),

$$\begin{aligned} \langle J_{\mathscr {B}} V\!J_{\mathscr {B}} \textbf{u}_{m}, \textbf{u}_{n}\rangle&= \langle J_{\mathscr {B}} V \textbf{u}_{m}, \textbf{u}_{n}\rangle \\&= \langle J_{\mathscr {B}} \textbf{u}_{n}, V \textbf{u}_{m}\rangle \\&= \langle \textbf{u}_n, V \textbf{u}_m\rangle \\&= \overline{\langle V \textbf{u}_{m}, \textbf{u}_{n}\rangle }. \end{aligned}$$

Thus, from our assumption that $[V]_{\mathscr {B}}^{t} = [V]_{\mathscr {B}}$, we see that

2.12 $$\begin{aligned}{}[J_{\mathscr {B}} V\!J_{\mathscr {B}}]_{\mathscr {B}} = \overline{[V]_{\mathscr {B}}} = \overline{[V]_{\mathscr {B}}^{t}} = [V^{*}]_{\mathscr {B}}. \end{aligned}$$

From (2.11) it follows that $[C^2]_{\mathscr {B}} = [I]_{\mathscr {B}}$ and so $C^2 = I$. Thus, *C* is a conjugation on $\mathcal {H}$.

### Remark 2.13

If *J* is *any* conjugation on $\mathcal {H}$, one can show there exists an orthonormal basis $\mathscr {B}$ for $\mathcal {H}$ such that $J = J_{\mathscr {B}}$ [18, Lemma 1]. Thus, there is some freedom in the above analysis, if so desired, to choose a conjugation *J* first instead of the orthonormal basis $\mathscr {B}$.

A version of the spectral theorem for unitary operators (see [7, Ch. IX, Thm. 2.2] or [33, Ch. 1]) says that if *U* is a unitary operator on $\mathcal {H}$, then there is a unique spectral measure $E(\cdot )$ defined on the Borel subsets of $\sigma (U)$, the spectrum of *U*, such that

2.14 $$\begin{aligned} U = \int _{\sigma (U)} \xi d E(\xi ). \end{aligned}$$

Moreover, for any spectral measure $E(\cdot )$ on $\mathbb {T}$ (i.e., $E(\cdot )$ is a projection-valued function on the Borel subsets of $\mathbb {T}$ such that $E(\mathbb {T}) = I$ and $E(\cdot )$ is countably additive), there is a unique unitary operator *U* associated with $E(\cdot )$ via (2.14).

For a spectral measure $E(\cdot )$ and $\textbf{x}, \textbf{y} \in \mathcal {H}$, the function $\mu _{\textbf{x}, \textbf{y}}(\cdot ):= \langle E(\cdot ) \textbf{x}, \textbf{y}\rangle$ defines a finite complex Borel measure on $\mathbb {T}$ and, in particular, for each $\textbf{x} \in \mathcal {H}$,

2.15 $$\begin{aligned} \mu _{\textbf{x}}:=\mu _{\textbf{x},\textbf{x}} \end{aligned}$$

defines a positive finite Borel measure on $\mathbb {T}$, sometimes called an *elementary measure*.

The following proposition from [24] describes the relationship between a unitary operator, its spectral measure, and a conjugation.

### Proposition 2.16

For a conjugation *C* and a unitary operator *U* on $\mathcal {H}$ with associated spectral measure $E(\cdot )$, the following are equivalent.

- $$C \in \mathscr {C}_{s}(U)$$;
- $C E(\Omega ) C = E(\Omega )$ for every Borel set $\Omega \subseteq \sigma (U)$.

## Decompositions of conjugations

As we will see in subsequent sections, the set $\mathscr {C}_s(U)$ is quite large and so an important step in understanding it is to decompose each $C \in \mathscr {C}_{s}(U)$ into more manageable pieces. This decomposition will involve various types of invariant subspaces. Recall that a (closed) subspace $\mathcal {M}$ of a Hilbert space $\mathcal {H}$ is *invariant* for $A \in \mathcal {B}(\mathcal {H})$ if $A \mathcal {M} \subseteq \mathcal {M}$; *reducing* if $A \mathcal {M} \subseteq \mathcal {M}$ and $A^{*} \mathcal {M} \subseteq \mathcal {M}$; and *hyperinvariant* if $T \mathcal {M} \subseteq \mathcal {M}$ for every $T \in \mathcal {B}(\mathcal {H})$ that commutes with *A*. We begin with the following lemma which follows from (2.1) and the fact that $C^2 = I$.

### Lemma 3.1

If *C* is a conjugation on $\mathcal {H}$ and $\mathcal {M}$ is a *C*-invariant subspace of $\mathcal {H}$, then $C \mathcal {M} = \mathcal {M}$ and $C\mathcal {M}^{\perp }=\mathcal {M}^\perp$.

Below is a useful decomposition theorem for a conjugation. For the rest of this paper, $M_{+}(\mathbb {T})$ will denote the set of all finite positive Borel measures on the unit circle $\mathbb {T}$. For $\mu , \sigma \in M_{+}(\mathbb {T})$ we use the standard notation $\mu \ll \sigma$ for $\mu$ is absolutely continuous with respect to $\sigma$ and $\mu \perp \sigma$ for $\mu$ is singular with respect to $\sigma$.

### Theorem 3.2

Let *U* be a unitary operator on $\mathcal {H}$, $E(\cdot )$ be its associated spectral measure, and for each $\textbf{x} \in \mathcal {H}$, let $\mu _{\textbf{x}}$ denote the associated elementary measure from (2.15). For any $\mu \in M_{+}(\mathbb {T})$, let

$$\begin{aligned} \mathcal {H}_\mu :=\{\textbf{x} \in \mathcal {H}: \mu _{\textbf{x}} \ll \mu \}. \end{aligned}$$

Then we have the following.

- $\mathcal {H}_\mu$ is a reducing subspace for *U*.
- If $$C \in \mathscr {C}_s(U)$$, then
  (i) $$C\mathcal {H}_\mu =\mathcal {H}_\mu $$ and $$C\mathcal {H}_\mu ^{\perp }=\mathcal {H}_\mu ^{\perp }$$.
  (ii) $C=C_\mu \oplus C_{\mu }^{\perp }$ where $C_\mu := C|_{\mathcal {H}_{\mu }}$ and $C_{\mu }^{\perp }: = C|_{\mathcal {H}_{\mu }^{\perp }}$.
  (iii) $$C_{\mu } (U|_{\mathcal {H}_{\mu }}) C_{\mu } = U^{*}|_{\mathcal {H}_{\mu }}$$ and $$C_{\mu }^{\perp } (U|_{\mathcal {H}_{\mu }^{\perp }} )C_{\mu }^{\perp } = U^{*}|_{\mathcal {H}_{\mu }^{\perp }}.$$

### Proof

The proof of (a) is routine and we omit it (see [25, §65, §66] for the details).

To prove (b), let $\textbf{x}\in \mathcal {H}_\mu$ and $\Omega$ be a Borel subset of $\sigma (U)$ for which $\mu (\Omega ) = 0$. By Proposition 2.16, *C* commutes with $E(\Omega )$ and thus, via (2.1),

$$\begin{aligned} \mu _{C \textbf{x}} = \langle E(\Omega )C\textbf{x},C\textbf{x}\rangle =\langle CE(\Omega )\textbf{x},C\textbf{x}\rangle = \langle \textbf{x},E(\Omega )\textbf{x}\rangle =\mu _{\textbf{x}}(\Omega ) = 0. \end{aligned}$$

This proves that $C\textbf{x}\in \mathcal {H}_\mu$. Since *C* is a conjugation, we see that $C\mathcal {H}_\mu =\mathcal {H}_\mu$ and $C\mathcal {H}_\mu ^\perp = \mathcal {H}_\mu ^\perp$ (Lemma 3.1). This proves (i) and (ii). Part (iii) follows from the facts that $\mathcal {H}_{\mu }$ is a reducing subspace for *U* and $C \in \mathscr {C}_{s}(U)$.

As a note here, other properties of the reducing subspace $\mathcal {H}_{\mu }$ were explored by Halmos in [25, §65]. Observe that if $\alpha \in \mathbb {T}$ and $\delta _{\alpha }$ denotes the point mass at $\alpha$, one can see that

$$\begin{aligned} \mathcal {H}_{\delta _{\alpha }} = \ker (U - \alpha I). \end{aligned}$$

### Corollary 3.3

For a unitary operator *U* on $\mathcal {H}$, let $C \in \mathscr {C}_s(U)$. Suppose $\alpha \in \mathbb {T}$. Then $C=C_{\delta _\alpha }\oplus C_{\delta _\alpha }^\perp ,$ where $C_{\delta _\alpha } \in \mathscr {C}_{s}(U|_{\ker (U - \alpha I)})$ and $C_{\delta _\alpha }^\perp \in \mathscr {C}_{s}(U|_{\ker (U-\alpha I)^\perp })$.

The following corollary will play an important role later on.

### Corollary 3.4

If *U* is a unitary operator on $\mathcal {H}$ with

$$\begin{aligned} \mathcal {H}= \bigoplus _{j \geqslant 1} \mathcal {E}_{\xi _j}, \quad \mathcal {E}_{\xi _j}:= \ker (U - \xi _{j} I),\end{aligned}$$

then

$$\begin{aligned} C = \bigoplus _{j \geqslant 1} C|_{\mathcal {E}_{\xi _j}} \end{aligned}$$

and

$$\begin{aligned} C|_{\mathscr {E}_{\xi _j}} U|_{\mathscr {E}_{\xi _j}} C|_{\mathscr {E}_{\xi _j}} = U^{*}|_{\mathscr {E}_{\xi _j}} \; \text{ for } \text{ all }\,j \geqslant 1. \end{aligned}$$

### Remark 3.5

Apply Theorem 3.2 to the case when $\mu = m$ (normalized Lebesgue measure on $\mathbb {T}$) and let $\mathcal {H}_{{\text {ac}}}: =\mathcal {H}_m$ and $\mathcal {H}_{{\text {sing}}}: =\mathcal {H}_{m}^\perp$. The decomposition $\mathcal {H} = \mathcal {H}_{{\text {ac}}} \oplus \mathcal {H}_{{\text {sing}}}$ was first explored in [25] and considered further in [3, 29, 30]. One can prove the following: Let *U* be a unitary operator on $\mathcal {H}$ and let $C \in \mathscr {C}_s(U)$. If $\mathcal {H}=\mathcal {H}_{{\text {ac}}}\oplus \mathcal {H}_{{\text {sing}}}$ is the Lebesgue decomposition from above, then $C=C_{{\text {ac}}}\oplus C_{{\text {sing}}}$, where $C_{{\text {ac}}} \in \mathscr {C}_{s}(U|_{\mathcal {H}_{{\text {ac}}}})$ and $C_{{\text {sing}}} \in \mathscr {C}_{s}(U|_{\mathcal {H}_{{\text {sing}}}})$.

A von Neumann–Wold type decomposition surveyed in [29] yields the following decomposition of a unitary operator *U* on $\mathcal {H}$ by reducing subspaces $\mathcal {H}_{{\text {ac}}}$, $\mathcal {L}$, and $\bigoplus _{\alpha \in \mathbb {T}} \ker (U - \alpha I)$ such that

3.6 $$\begin{aligned} \mathcal {H} = \mathcal {H}_{{\text {ac}}} \bigoplus \mathcal {L} \bigoplus _{\alpha \in \mathbb {T}} \ker (U - \alpha I). \end{aligned}$$

Here one proves the following: Let *U* be a unitary operator on $\mathcal {H}$ and $C \in \mathscr {C}_s(U)$. If $\mathcal {H}$ is decomposed as in (3.6), then there is an appropriate decomposition of *C* as $C=C_{ac}\oplus C|_{\mathcal {L}}\oplus \bigoplus _{\alpha \in \mathbb {T}} C_{\delta _\alpha }$, where each summand is a conjugation on the corresponding space.

Though Theorem 3.2 seems relatively simple, it yields interesting results about hyperinvariant subspaces of unitary operators. For a unitary operator, every hyperinvariant subspace is also reducing (but not the converse as one can see with a diagonal matrix). Theorem 3.8 below connects hyperinvariant subspaces with conjugations. The culmination of this discussion will be given in Theorems 8.6 and 8.8.

### Theorem 3.7

Let *U* be a unitary operator on $\mathcal {H}$ with spectral measure $E(\cdot )$. Then every hyperinvariant subspace for *U* is invariant for every $C \in \mathscr {C}_{s}(U)$. Moreover, every hyperinvariant subspaces can be written as $E(\Omega )\mathcal {H}$, where $\Omega$ is a Borel subset of $\sigma (U)$.

### Proof

From [33, Proposition 6.9] (see also [8]), every hyperinvariant subspace for *U* can be written as $E(\Omega )\mathcal {H}$ for some Borel set $\Omega \subseteq \sigma (U)$. Since *C* commutes with $E(\cdot )$ (Proposition 2.16(b)), $E(\Omega )\mathcal {H}$ is invariant for *C*.

We now connect $\mathcal {H}_{\mu }$ with hyperinvariant subspaces. A first step in this direction is this following result – which might be contained somewhere in the literature but we include a proof for the reader’s convenience. We will see an extension of this result in Theorems 8.6 and 8.8 below.

### Theorem 3.8

If $\mathcal {M}$ is a hyperinvariant subspace of a unitary operator *U* on $\mathcal {H}$, then there is a $\mu \in M_{+}(\mathbb {T})$ for which $\mathcal {M} = \mathcal {H}_{\mu }$.

### Proof

From Theorem 3.7, every hyperinvarient subspace $\mathcal {M}$ of *U*, with associated spectral measure $E(\cdot )$, takes the form $\mathcal {M} = E(\Omega ) \mathcal {H}$ for some Borel subset $\Omega \subseteq \sigma (U)$. Since $E(\Omega )\mathcal {H}$ is also reducing, then $V:= U|_{E(\Omega ) \mathcal {H}}$ is a unitary operator whose spectral measure is $E(\cdot )E(\Omega )$.

Let $\mathcal {W}^{*}(V)$ denote the von Neumann algebra generated by *V* (i.e., the smallest $*$-closed and strongly closed algebra containing *V*). Using [7, Ch. IX, Cor. 7.9] one can produce a *separating vector* $\textbf{e} \in \mathcal {H}$ for $\mathcal {W}^{*}(V)$ in that if $A \in \mathcal {W}^{*}(V)$ satisfies $A \textbf{e} = \textbf{0}$, then $A \equiv 0$. By [7, Ch. IX, Prop. 8.3], the corresponding elementary measure $\mu _{\textbf{e}}$ from (2.15) is a *scalar-valued spectral measure* for *V* in that $\mu _{\textbf{e}}(\Delta ) = 0$ if and only if $E(\Delta ) E(\Omega ) = E(\Delta \cap \Omega )= 0$.

To complete the proof, we will show that $E(\Omega ) \mathcal {H}= \mathcal {H}_{\mu _{\textbf{e}}}$. For the $\subseteq$ containment, observe that if $\textbf{x} \in E(\Omega ) \mathcal {H}$ and $\Delta$ is a Borel subset of $\Omega$, then

3.9 $$\begin{aligned} \mu _{\textbf{x}}(\Delta ) = \langle E(\Delta ) \textbf{x}, \textbf{x}\rangle = \Vert E(\Delta ) \textbf{x}\Vert ^2 \end{aligned}$$

and thus, since $\mu _{\textbf{e}}$ is a scalar valued spectral measure for $V = U|_{E(\Omega ) \mathcal {H}}$, we see that if $\mu _{\textbf{e}}(\Delta ) = 0$, then $E(\Delta ) = 0$. Thus, by (3.9), $\mu _{\textbf{x}} \ll \mu _{\textbf{e}}$ and so $\textbf{x} \in \mathcal {H}_{\mu _{\textbf{e}}}$. This verifies $E(\Omega ) \mathcal {H}\subseteq \mathcal {H}_{\mu _{\textbf{e}}}$.

For the $\supseteq$ containment, let $\textbf{y} \in \mathcal {H}_{\mu _{\textbf{e}}}$. Then $\mu _{\textbf{y}} \ll \mu _{\textbf{e}}$ and so $\mu _{\textbf{y}}(\Delta ) = 0$ whenever $\mu _{\textbf{e}}(\Delta ) = 0$. We want to show that $\textbf{y} \in E(\Omega ) \mathcal {H}$. Since $\mu _{\textbf{e}}$ is a scalar valued spectral measure for *V*, we see that $\mu _{\textbf{e}}(\sigma (U) \setminus \Omega ) = 0$. Using the assumption that $\mu _{\textbf{y}} \ll \mu _{\textbf{e}}$, we obtain

$$\begin{aligned} \mu _{\textbf{y}}(\sigma (U) \setminus \Omega ) = \Vert E(\sigma (U) \setminus \Omega ) \textbf{y}\Vert ^2 = 0. \end{aligned}$$

Thus, since $\mathcal {H}= (E(\Omega )\mathcal {H}) \oplus (E(\sigma (U) {\setminus } \Omega )\mathcal {H})$, it follows that $\textbf{y} \in E(\Omega ) \mathcal {H}$. This verifies $\mathcal {H}_{\mu _{\textbf{e}}} \subseteq E(\Omega ) \mathcal {H}$.

We end this section with a discussion of when $\mathcal {H}_{\nu _1} \subseteq \mathcal {H}_{\nu _2}$ for $\nu _1, \nu _2 \in M_{+}(\mathbb {T})$. Observe that if $\nu$ is a scalar spectral measure for *U* (i.e., $\nu (\Delta ) = 0$ if and only if $E(\Delta ) = 0$) then $\mathcal {H}_\nu =\mathcal {H}$. Next let

$$\begin{aligned}{}[\textbf{x}]_{U, U^{*}}:= \bigvee \{ U^{n} \textbf{x}: n \in \mathbb {Z}\}, \end{aligned}$$

where $\bigvee$ denotes the closed linear span, denote the $*$-cyclic invariant subspace generated by $\textbf{x}$. For any $\mu \in M_{+}(\mathbb {T})$ the condition $\textbf{x}\in \mathcal {H}_\mu$ implies that $[\textbf{x}]_{U, U^{*}}\subseteq \mathcal {H}_\mu$ (since $\mathcal {H}_{\mu }$ is reducing).

Recall [25, §48] the standard Boolean operations $\wedge$ and $\vee$ for $\mu _1, \mu _2 \in M_{+}(\mathbb {T})$ defined on Borel subsets $\Omega$ of $\mathbb {T}$ by

$$\begin{aligned}{} & {} (\mu _1 \vee \mu _2) (\Omega ) = \mu _1(\Omega ) + \mu _2(\Omega );\\{} & {} (\mu _1 \wedge \mu _2)(\Omega ) = \inf \{\mu _1(\Omega \cap A) + \mu _2(\Omega \setminus A): A\hbox { is a Borel set}\}. \end{aligned}$$

### Proposition 3.10

Let *U* be a unitary operator on $\mathcal {H}$ and $\mu$ be any scalar spectral measure for *U*. For $\nu _1, \nu _2 \in M_{+}(\mathbb {T})$ the following are equivalent.

- $$\mathcal {H}_{\nu _1}\subseteq \mathcal {H}_{\nu _2};$$
- $$\nu _1\wedge \mu \ll \nu _2\wedge \mu .$$

### Proof

Assume condition (b) and let $\textbf{x}\in \mathcal {H}_{\nu _1}$. Then $\langle E(\cdot )\textbf{x},\textbf{x}\rangle \ll \nu _1$. Since $\mu$ is a scalar spectral measure for *U*, we see that $\langle E(\cdot )\textbf{x},\textbf{x}\rangle \ll \mu$. Hence $\langle E(\cdot )\textbf{x},\textbf{x}\rangle \ll \nu _1\wedge \mu \ll \nu _2\wedge \mu$. Thus $\langle E(\cdot )\textbf{x},\textbf{x}\rangle \ll \nu _2$ and so $\textbf{x}\in \mathcal {H}_{\nu _2}$. Thus, $(b) \Longrightarrow (a)$.

For the proof of $(a) \Longrightarrow (b)$, let us assume that $\nu _1\wedge \mu$ is not absolutely continuous with respect to $\nu _2\wedge \mu$. Then there is a nonzero measure $\nu \in M_{+}(\mathbb {T})$ such that $\nu \ll (\nu _1\wedge \mu )$ and $\nu \perp (\nu _2\wedge \mu )$. Let $\textbf{e}\in \mathcal {H}$ be a vector such that $\mu _{\textbf{e}}$ is a scalar spectral measure (for example a separating vector for von Neumann algebra generated by *U* – from the proof of Theorem 3.8). Since $\mu$ and $\mu _{\textbf{e}}$ are mutually absolutely continuous as scalar spectral measures for *U*, it follows that $\nu \ll \mu _{\textbf{e}}$. By [7, Ch.IX, Lemma 8.6] there is a nonzero $\textbf{y}\in [\textbf{x}]_{U, U^{*}}$ such that $\mu _{\textbf{y}}=\nu$. Therefore, $\textbf{y}\in \mathcal {H}_{\nu _1}$. On the other hand if $\textbf{y}\in \mathcal {H}_{\nu _2}$ then $\mu _{\textbf{y}}=\nu \ll \nu _2\wedge \mu$, which is a contradiction.

### Corollary 3.11

Let $\nu _1, \nu _2 \in M_{+}(\mathbb {T})$. If $\nu _1 \ll \nu _2$ then $\mathcal {H}_{\nu _1} \subseteq \mathcal {H}_{\nu _2}$.

### Corollary 3.12

Let *U* be a unitary operator on $\mathcal {H}$ and $\mu$ be any scalar spectral measure for *U*. For $\nu _1, \nu _2 \in M_{+}(\mathbb {T})$ the following are equivalent.

- $$\mathcal {H}_{\nu _1}= \mathcal {H}_{\nu _2}$$;
- $\nu _1\wedge \mu$ and $\nu _2\wedge \mu$ are mutually absolutely continuous.

## A matrix description of $\mathscr {C}_s(U)$

Our first description of $\mathscr {C}_s(U)$ will involve matrix a representation of *U* with respect to an orthonormal basis. Though this might seem a bit difficult to apply at first, we will see, through our decomposition theorem from the previous section, that it can often yield a tangible description of $\mathscr {C}_s(U)$. Recall our notation from §2, where, for a fixed orthonormal basis $\mathscr {B} = \{\textbf{u}_{j}\}_{j \geqslant 1}$ for a (separable) Hilbert space $\mathcal {H}$ and for $A \in \mathcal {B}(\mathcal {H})$, $[A]_{\mathscr {B}}$ denotes the matrix representation of *A* with respect to $\mathscr {B}$, and $J_{\mathscr {B}}$ denotes the conjugation on $\mathcal {H}$ for which $J_{\mathscr {B}} \textbf{u}_n = \textbf{u}_{n}$ for all $n \geqslant 1$.

### Theorem 4.1

Suppose *U* is a unitary operator on $\mathcal {H}$. Then $C \in \mathscr {C}_s(U)$ if and only if there is a unitary operator *V* on $\mathcal {H}$ satisfying

- $$[V]_{\mathscr {B}}^{t} = [V]_{\mathscr {B}}$$;
- $$[V]_{\mathscr {B}} [U]_{\mathscr {B}}^{t} = [U]_{\mathscr {B}} [V]_{\mathscr {B}}$$;
- $$C = V\!J_{\mathscr {B}}$$.

### Proof

Proposition 2.10 says that *C* is a conjugation on $\mathcal {H}$ if and only if $C = V\!J_{\mathscr {B}}$ for some unitary *V* on $\mathcal {H}$ with $[V]_{\mathscr {B}}^{t} = [V]_{\mathscr {B}}$. Now, to fulfill the requirement that $C \in \mathscr {C}_{s}(U)$, observe that

$$\begin{aligned} C U^{*} C = U&\iff (V\!J_{\mathscr {B}}) U^{*} (V\!J_{\mathscr {B}} ) = U\\&\iff V (J_{\mathscr {B}} U^{*}\!J_{\mathscr {B}}) (J_{\mathscr {B}} V\!J_{\mathscr {B}}) = U\\&\iff [V]_{\mathscr {B}} [J_{\mathscr {B}} U^{*}\!J_{\mathscr {B}}]_{\mathscr {B}} [J_{\mathscr {B}} V \!J_{\mathscr {B}} ]_{\mathscr {B}} = [U]_{\mathscr {B}}\\&\iff [V]_{\mathscr {B}} [U]_{\mathscr {B}}^{t} [V^{*}]_{\mathscr {B}} = [U]_{\mathscr {B}}. \end{aligned}$$

Notice the use of (2.12) above. Thus,

$$\begin{aligned} C U^{*} C = U \iff [V]_{\mathscr {B}} [U]_{\mathscr {B}}^{t} = [U]_{\mathscr {B}} [V]_{\mathscr {B}}, \end{aligned}$$

which completes the proof.

One can use the above discussion to describe $\mathscr {C}_s(U)$ when *U* is an $n \times n$ unitary matrix.

### Theorem 4.2

Suppose *U* is an $n \times n$ unitary matrix with spectral decomposition

$$\begin{aligned}U = W \begin{bmatrix} \xi _1 I_{n_1} &{} &{} &{} \\ &{} \xi _2 I_{n_2} &{} &{} \\ &{} &{} \ddots &{} \\ &{} &{} &{} \xi _d I_{n_d} \end{bmatrix} W^{*},\end{aligned}$$

where *W* is an $n \times n$ unitary matrix, $\xi _1, \ldots , \xi _d \in \mathbb {T}$ are the distinct eigenvalues of *U*, $I_{n_j}$ is the $n_j \times n_j$ identity matrix, and $n_1 + n_2 + \cdots + n_d = n$. Then $C \in \mathscr {C}_{s}(U)$ if and only if

4.3 $$\begin{aligned} C = W \begin{bmatrix} V_1 &{} &{} &{} \\ &{} V_2 &{} &{} \\ &{} &{} \ddots &{}\\ &{} &{} &{} V_d \end{bmatrix} J W^{*}, \end{aligned}$$

where, for each $1 \leqslant j \leqslant d$, $V_j$ is an $n_j \times n_j$ unitary matrix which satisfies $V_{j}^{t} = V_j$, and *J* is the conjugation on $\mathbb {C}^n$ defined by

$$\begin{aligned} J[x_1 \; x_2 \; \cdots \; x_n]^{t} = [\overline{x_1} \; \overline{x_2} \; \cdots \; \overline{x_n}]^{t}. \end{aligned}$$

### Proof

Suppose that $C \in \mathscr {C}_{s}(U)$. For the unitary matrix

$$\begin{aligned}\widetilde{U} = \begin{bmatrix} \xi _1 I_{n_1} &{} &{} &{} \\ &{} \xi _2 I_{n_2} &{} &{} \\ &{} &{} \ddots &{} \\ &{} &{} &{} \xi _d I_{n_d} \end{bmatrix}, \end{aligned}$$

Corollary 3.4 says that any $\widetilde{C} \in \mathscr {C}_{s}(\widetilde{U})$ can be written as

$$\begin{aligned} \widetilde{C} = \widetilde{C}|_{\mathcal {E}_{\xi _{1}}} \oplus \widetilde{C}|_{\mathcal {E}_{\xi _{2}}} \oplus \cdots \oplus \widetilde{C}|_{\mathcal {E}_{\xi _{d}}}, \end{aligned}$$

where

$$\begin{aligned} \mathscr {E}_{j} = \ker (\widetilde{U} - \xi _j I) \end{aligned}$$

(which is the span of appropriate standard basis vectors for $\mathbb {C}^n$) and

$$\begin{aligned} \widetilde{C}_{j}:= \widetilde{C}|_{\mathcal {E}_{\xi }} \in \mathscr {C}_s( \widetilde{U}|_{\mathscr {E}_{\xi _j}}) \; \text{ for } \text{ all }\,1 \leqslant j \leqslant d. \end{aligned}$$

Theorem 4.1 says that for each $1 \leqslant j \leqslant d$ there is an $n_j \times n_j$ unitary matrix $V_j$ such that such that $\widetilde{C}_{j} = V_{j} J|_{\mathscr {E}_{j}}$ and $V_{j}^{t} = V_j$. Note that $J|_{\mathscr {E}_j}$ fixes the appropriate standard basis vectors. Since $\widetilde{U}|_{\mathcal {E}_{\xi _j}} = \xi _j I_{n_j}$, condition (b) of Theorem 4.1 is automatic. Thus, every $\widetilde{C} \in \mathscr {C}_{s}(\widetilde{U})$ takes the form

$$\begin{aligned} \begin{bmatrix} V_1 &{} &{} &{} \\ &{} V_2 &{} &{} \\ &{} &{} \ddots &{}\\ &{} &{} &{} V_d \end{bmatrix} J.\end{aligned}$$

Now apply Lemma 2.3 and Proposition 2.6 to see that *C* takes the form in (4.3).

Conversely suppose that *C* is the conjugation from (4.3). Then, due to the fact that each of the unitary matrices $V_j$ satisfy $V_{j}^{t} = V_j$ and $J A J = \overline{A}$ for any matrix *A*, *CUC* is equal to

$$\begin{aligned}&W \begin{bmatrix} V_1 &{} &{} &{} \\ &{} V_2 &{} &{} \\ &{} &{} \ddots &{}\\ &{} &{} &{} V_d \end{bmatrix} J \begin{bmatrix} \xi _1 I_{n_1} &{} &{} &{} \\ &{} \xi _2 I_{n_2} &{} &{} \\ &{} &{} \ddots &{} \\ &{} &{} &{} \xi _d I_{n_d} \end{bmatrix} \begin{bmatrix} V_1 &{} &{} &{} \\ &{} V_2 &{} &{} \\ &{} &{} \ddots &{}\\ &{} &{} &{} V_d \end{bmatrix} J W^{*} \\&= W \begin{bmatrix} V_1 &{} &{} &{} \\ &{} V_2 &{} &{} \\ &{} &{} \ddots &{}\\ &{} &{} &{} V_d \end{bmatrix} J \begin{bmatrix} \xi _1 V_1 &{} &{} &{} \\ &{} \xi _2 V_2 &{} &{} \\ &{} &{} \ddots &{} \\ &{} &{} &{} \xi _d V_d \end{bmatrix} J W^{*}\\&= W \begin{bmatrix} V_1 &{} &{} &{} \\ &{} V_2 &{} &{} \\ &{} &{} \ddots &{}\\ &{} &{} &{} V_d \end{bmatrix} \begin{bmatrix} \overline{\xi _1} \overline{V_1} &{} &{} &{} \\ &{}\overline{ \xi _2} \overline{ V_2} &{} &{} \\ &{} &{} \ddots &{} \\ &{} &{} &{} \overline{ \xi _d} \overline{ V_d} \end{bmatrix} W^{*}\\&= W \begin{bmatrix} V_1 &{} &{} &{} \\ &{} V_2 &{} &{} \\ &{} &{} \ddots &{}\\ &{} &{} &{} V_d \end{bmatrix} \begin{bmatrix} \overline{\xi _1} V_{1}^{*} &{} &{} &{} \\ &{}\overline{ \xi _2} V_{2}^{*} &{} &{} \\ &{} &{} \ddots &{} \\ &{} &{} &{} \overline{ \xi _d} V_{d}^{*} \end{bmatrix} W^{*}\\&= W \begin{bmatrix} \overline{\xi _1} I_{n_1} &{} &{} &{} \\ &{}\overline{ \xi _2} I_{n_2} &{} &{} \\ &{} &{} \ddots &{} \\ &{} &{} &{} \overline{ \xi _d} I_{d_d} \end{bmatrix} W^{*} = U^{*}. \end{aligned}$$

Thus $C \in \mathscr {C}_{c}(U)$, which completes the proof.

In certain circumstances, we can extend Theorem 4.2 to the infinite dimensional setting. We mention two interesting examples of unitary operators from harmonic analysis.

### Example 4.4

Let

$$\begin{aligned} (\mathcal {F}f)(x) = \frac{1}{\sqrt{2 \pi }} \int _{-\infty }^{\infty } f(t) e^{-ixt} dt, \end{aligned}$$

denote the classical Fourier–Plancherel transform on $L^2(\mathbb {R})$ (defined initially on $L^1(\mathbb {R}) \cap L^2(\mathbb {R})$ by the above integral and extended to be a unitary operator on $L^2(\mathbb {R})$). It is known that $\sigma (\mathcal {F}) = \sigma _{p}(\mathcal {F}) = \{1, -1, i, -i\}$ and $\mathcal {F} H_n = (-i)^n H_n$, where $H_n$ is the *n*th Hermite function, i.e.,

$$\begin{aligned} H_{n}(x) = c_n e^{-x^2/2} h_{n}(x), \; \; n \geqslant 0, \end{aligned}$$

$c_n$ is a constant for which $\Vert H_n\Vert _{L^2(\mathbb {R})}= 1$, and $h_n$ is the *n*-th Hermite polynomial. Moreover, $\mathscr {B} = \{H_{n}\}_{n \geqslant 0}$ forms an orthonormal basis for $L^2(\mathbb {R})$ [16, Ch.11]. Thus, using our earlier notation from Corollary 3.4,

4.5 $$\begin{aligned} L^2(\mathbb {R}) = \mathcal {E}_{1} \bigoplus \mathcal {E}_{-i} \bigoplus \mathcal {E}_{-1} \bigoplus \mathcal {E}_{i}, \end{aligned}$$

where $\mathscr {B}_{1} = \{H_{4 k}\}_{k \geqslant 0},$ $\mathscr {B}_{-i} = \{H_{4 k + 1}\}_{k \geqslant 0},$ $\mathscr {B}_{-1} = \{H_{4 k + 2}\}_{k \geqslant 0},$ $\mathscr {B}_{i} = \{H_{4 k + 3}\}_{k \geqslant 0},$ are orthonormal bases for $\mathcal {E}_{1}, \mathcal {E}_{-i}, \mathcal {E}_{-1}, \mathcal {E}_{-i}$ respectively, we can write $\mathcal {F}$ in block operator form with respect to the decomposition in (4.5) as

$$\begin{aligned}\mathcal {F} = \begin{bmatrix} I|_{\mathcal {E}_{1}} &{} &{} &{} \\ &{} -i I|_{\mathcal {E}_{-i}} &{} &{} \\ &{} &{} -I|_{\mathcal {E}_{-1}} &{} \\ &{} &{} &{} i I|_{\mathcal {E}_{i}} \end{bmatrix}.\end{aligned}$$

Furthermore, any conjugation *C* on $L^2(\mathbb {R})$ for which $C \mathcal {F} C = \mathcal {F}^{*}$ must take the (block) form

$$\begin{aligned}C = \begin{bmatrix} V_1 &{} &{} &{}\\ &{} V_{-i} &{} &{}\\ &{} &{} V_{-1} &{}\\ &{} &{} &{} V_i\\ \end{bmatrix} \begin{bmatrix} J_1 &{} &{} &{}\\ &{} J_{-i} &{} &{}\\ &{} &{} J_{-1} &{}\\ &{} &{} &{} J_{i}\\ \end{bmatrix},\end{aligned}$$

where, for each $k \in \{1, -i, -1, i\}$, $J_{k}$ is the conjugation on $\mathcal {E}_{k}$ which fixes every element of $\mathscr {B}_{k}$ and $V_{k}$ is a unitary operator on $\mathcal {E}_{k}$ for which $[V_k]_{\mathscr {B}_k}^{t} = [V_k]_{\mathscr {B}_k}$.

### Example 4.6

Suppose

$$\begin{aligned} (\mathscr {H} g)(x) = \frac{1}{\pi } {\text {PV}} \int _{-\infty }^{\infty } \frac{g(t)}{x - t} dt, \end{aligned}$$

is the Hilbert transform on $L^2(\mathbb {R})$. Then $\sigma (\mathscr {H}) = \sigma _{p}(\mathscr {H}) = \{i, -i\}$ and, again using the notation from Corollary 3.4, $L^2(\mathbb {R}) = \mathcal {E}_{i} \bigoplus \mathcal {E}_{-i}.$ Moreover, $\mathcal {E}_{i}$ has orthonormal basis $\mathscr {B}_{i} = \{f_n\}_{n \geqslant 1}$, where

$$\begin{aligned} f_n(x) = \frac{1}{\sqrt{\pi }} \frac{(x + i)^{n - 1}}{(x - i)^{n}}, \end{aligned}$$

and $\mathcal {E}_{-i}$ has orthonormal basis $\mathscr {B}_{-i} = \{g_n\}_{n \geqslant 0}$, where

$$\begin{aligned} g_{n}(x) = \frac{1}{\sqrt{\pi }} \frac{(x - i)^{n}}{(x + i)^{n + 1}}. \end{aligned}$$

See [16, Ch. 12] for the details. Moreover, by a similar discussion as in the previous example, any conjugation *C* on $L^2(\mathbb {R})$ for which $C \mathscr {H} C = \mathscr {H}^{*}$ must take the (block) form

$$\begin{aligned}C = \begin{bmatrix} V_i &{} \\ &{} V_{-i} \end{bmatrix} \begin{bmatrix} J_{i} &{} \\ &{} J_{-i} \end{bmatrix},\end{aligned}$$

where for each $k \in \{i, -i\}$, $J_{k}$ is the conjugation on $\mathcal {E}_{k}$ which fixes every element of $\mathscr {B}_{i}$ and $V_{k}$ is a unitary operator on $\mathcal {E}_{k}$ for which $[V_k]_{\mathscr {B}_{k}}^{t} = [V_k]_{\mathscr {B}_{k}}$.

## Natural conjugations on vector valued $L^2$ spaces

This section provides a model for conjugations on vector valued Lebesgue spaces. It will be useful for our description of $\mathscr {C}_s(U)$ in §8 and also sets up our discussion of models for bilateral shifts, and their associated conjugations, in the next section.

For a Hilbert space $\mathcal {H}$ with norm $\Vert \cdot \Vert _{\mathcal {H}}$ and $\mu \in M_{+}(\mathbb {T})$, consider the set $\mathscr {L}^{0}(\mu , \mathcal {H})$ of all $\mathcal {H}$-valued $\mu$-measurable functions ${{\varvec{f}}}$ on $\mathbb {T}$ and the Hilbert space

$$\begin{aligned} \mathscr {L}^{2}(\mu , \mathcal {H}):= \Big \{{{\varvec{f}}}\in \mathscr {L}^{0}(\mu , \mathcal {H}): \Vert {{\varvec{f}}}\Vert _{L^2(\mu . \mathcal {H})}:= \Big (\int _{\mathbb {T}} \Vert {{\varvec{f}}}(\xi )\Vert ^{2}_{\mathcal {H}}d\mu (\xi )\Big )^{1/2} < \infty \Big \}. \end{aligned}$$

One often sees this using tensor notation as $L^2(\mu ) \otimes \mathcal {H}$. Also consider $\mathscr {L}^{\infty }(\mu , \mathcal {B}(\mathcal {H}))$, the $\mu$-essentially bounded $\mathcal {B}(\mathcal {H})$-valued functions $\textbf{U}$ on $\mathbb {T}$. For $\textbf{U}\in \mathscr {L}^{\infty }(\mu , \mathcal {B}(\mathcal {H}))$, define the multiplication operator ${{\varvec{M}}}_{\textbf{U}}$ on $\mathscr {L}^2(\mu , \mathcal {H})$ by

5.1 $$\begin{aligned} ({{\varvec{M}}}_{\textbf{U}}\,{{\varvec{f}}})(\xi )=\textbf{U}(\xi ){{\varvec{f}}}(\xi ) \end{aligned}$$

for ${{\varvec{f}}}\in \mathscr {L}^2(\mu ,\mathcal {H})$ and $\mu$-almost every $\xi \in \mathbb {T}$. Clearly ${{\varvec{M}}}_{\textbf{U}}\in \mathcal {B}(\mathscr {L}^2(\mu ,\mathcal {H}))$. If we use the notation $\textbf{U}^{*}(\xi ) = \textbf{U}(\xi )^{*}$, one can verify that

$$\begin{aligned} {{\varvec{M}}}_{\textbf{U}}^{*} = {{\varvec{M}}}_{\textbf{U}^{*}}. \end{aligned}$$

We let $L^{\infty }(\mu ):= \mathscr {L}^{\infty }(\mu , \mathbb {C})$ to denote the set of all scalar valued $\mu$-essentially bounded functions on $\mathbb {T}$. For ease of notation, we will write ${{\varvec{M}}}_{\varphi }$, when $\varphi \in L^{\infty }(\mu )$, in place of the more cumbersome ${{\varvec{M}}}_{\varphi {{\varvec{I}}}_{\mathcal {H}}}$, that is,

5.2 $$\begin{aligned} ({{\varvec{M}}}_\varphi {{\varvec{f}}})(\xi )=({{\varvec{M}}}_{\varphi {{\varvec{I}}}_\mathcal {H}}{{\varvec{f}}})(\xi )=\varphi (\xi ){{\varvec{f}}}(\xi ) \end{aligned}$$

for ${{\varvec{f}}}\in \mathscr {L}^2(\mu , \mathcal {H})$ and for $\mu$-almost every $\xi \in \mathbb {T}$. Of particular importance is when $\varphi (\xi ) = \xi$ which yields the (bilateral) shift ${{\varvec{M}}}_{\xi }$ on $\mathscr {L}^2(\mu , \mathcal {H})$. As a specific example, we have *the* bilateral shift $(M_{\xi } f)(\xi ) = \xi f(\xi )$ on $L^2(m, \mathbb {T})$ when $\mathcal {H}= \mathbb {C}$.

Recall from §2 that $\mathscr {A}\!\mathcal {B}(\mathcal {H})$ denotes the space of all bounded antilinear operators on $\mathcal {H}$. By $\mathscr {L}^{\infty }(\mu , \mathscr {A}\!\mathcal {B}(\mathcal {H}))$ we denote the space of all $\mu$-essentially bounded and $\mathscr {A}\!\mathcal {B}(\mathcal {H})$-valued Borel functions on $\mathbb {T}$. Analogously, as in (5.1), for $\textbf{C}\in \mathscr {L}^{\infty }(\mu , \mathscr {A}\!\mathcal {B}(\mathcal {H}))$, define

$$\begin{aligned} ({{\varvec{A}}}_\textbf{C}{{\varvec{f}}})(\xi )=\textbf{C}(\xi ){{\varvec{f}}}(\xi ) \end{aligned}$$

for ${{\varvec{f}}}\in \mathscr {L}^2(\mu ,\mathcal {H})$ and for $\mu$-almost every $\xi \in \mathbb {T}$. One can check that ${{\varvec{A}}}_\textbf{C}\in \mathscr {A}\!\mathcal {B}(\mathscr {L}^2(\mu ,\mathcal {H}))$.

For any conjugation *J* on $\mathcal {H}$, define the associated conjugation ${{\varvec{J}}}$ on $\mathscr {L}^2(\mu ,\mathcal {H})$ by

5.3 $$\begin{aligned} ({{\varvec{J}}}{{\varvec{f}}})(\xi )=J({{\varvec{f}}}(\xi )). \end{aligned}$$

For example, when $\mathcal {H}= \mathbb {C}$ and $J z = \overline{z}$ on $\mathbb {C}$, then ${{\varvec{J}}}$ is the conjugation on $L^2(\mu )$ defined by ${{\varvec{J}}}f = \overline{f}$ and discussed earlier. One can check that for ${{\varvec{f}}}\in \mathscr {L}^2(\mu , \mathcal {H})$ and $\mu$-almost every $\xi \in \mathbb {T}$ we have

$$\begin{aligned} ({{\varvec{J}}}{{\varvec{M}}}_{\xi } {{\varvec{J}}}{{\varvec{f}}})(\xi ) = ({{\varvec{M}}}_{\bar{\xi }} {{\varvec{f}}})(\xi ) \end{aligned}$$

and thus,

5.4 $$\begin{aligned} {{\varvec{J}}}{{\varvec{M}}}_\xi {{\varvec{J}}}={{\varvec{M}}}_{\bar{\xi }}. \end{aligned}$$

Proposition 5.6 below echos a result from [5, Proposition 4.2] and relies on the following (easily verified) lemma.

### Lemma 5.5

Let *U* be a unitary operator and *C* be a conjugation on $\mathcal {H}$. Then *UC* is a conjugation on $\mathcal {H}$ if and only if $C \in \mathscr {C}_{s}(U)$.

### Proposition 5.6

Let *J* be a conjugation on $\mathcal {H}$, ${{\varvec{J}}}$ be defined by (5.3), and let $\textbf{U}\in \mathscr {L}^{\infty }(\mu , \mathcal {B}(\mathcal {H}))$ be such that $\textbf{U}(\xi )$ is unitary for $\mu$-almost every $\xi \in \mathbb {T}$. Then the following hold.

- ${{\varvec{M}}}_\textbf{U}{{\varvec{J}}}$ is a conjugation on $\mathscr {L}^2(\mu , \mathcal {H})$ if and only if $\textbf{U}(\xi )$ is *J*–symmetric for $\mu$-almost every $\xi \in \mathbb {T}$.
- When ${{\varvec{M}}}_\textbf{U}{{\varvec{J}}}$ is a conjugation on $\mathscr {L}^2(\mu , \mathcal {H})$, we have $({{\varvec{M}}}_\textbf{U}{{\varvec{J}}}){{\varvec{M}}}_\xi ({{\varvec{M}}}_\textbf{U}{{\varvec{J}}})={{\varvec{M}}}_{\bar{\xi }}$.

### Proof

The proofs that the mapping ${{\varvec{M}}}_{\textbf{U}}{{\varvec{J}}}$ is antilinear and isometric on the space $\mathscr {L}^2(\mu , \mathcal {H})$ are straightforward. Assume that ${{\varvec{M}}}_{\textbf{U}} {{\varvec{J}}}$ is a conjugation. It follows from Lemma 5.5 that for every ${{\varvec{f}}}\in \mathscr {L}^2(\mu , \mathcal {H})$,

$$\begin{aligned} ({{\varvec{M}}}_{\textbf{U}} {{\varvec{J}}}{{\varvec{f}}}) (\xi ) = \textbf{U}(\xi ) ({{\varvec{J}}}{{\varvec{f}}})(\xi ) = \textbf{U}(\xi ) J({{\varvec{f}}}(\xi )) \end{aligned}$$

must be equal to

$$\begin{aligned} ({{\varvec{J}}}{{\varvec{M}}}_{\textbf{U}^{*}} {{\varvec{f}}})(\xi ) = J(({{\varvec{M}}}_{\textbf{U}^{*}}{{\varvec{f}}})(\xi )) = J((\textbf{U}^{*}(\xi ) {{\varvec{f}}})(\xi )). \end{aligned}$$

Therefore, $\textbf{U}(\xi ) J = J \textbf{U}^{*}(\xi )$ for $\mu$-almost every $\xi \in \mathbb {T}$ and thus, $\textbf{U}(\xi )$ is *J*-symmetric for $\mu$-almost every $\xi \in \mathbb {T}$. The above argument can be reversed, which proves (a).

To prove (b), observe that for any ${{\varvec{f}}}\in \mathscr {L}^2(\mu , \mathcal {H})$ we use the fact that $J \textbf{U}(\xi ) J = \textbf{U}^{*}(\xi )$ for $\mu$-almost every $\xi \in \mathbb {T}$ to see that

$$\begin{aligned} ({{\varvec{M}}}_{\textbf{U}} {{\varvec{J}}}) {{\varvec{M}}}_{\xi } ({{\varvec{M}}}_{\textbf{U}} {{\varvec{J}}}{{\varvec{f}}})(\xi )&= \textbf{U}(\xi ) ({{\varvec{J}}}{{\varvec{M}}}_{\xi } {{\varvec{M}}}_{\textbf{U}} {{\varvec{J}}}{{\varvec{f}}})(\xi )\\&= \textbf{U}(\xi ) J (({{\varvec{M}}}_{\xi } {{\varvec{M}}}_{\textbf{U}}{{\varvec{J}}}{{\varvec{f}}})(\xi ))\\&= \textbf{U}(\xi ) J((\xi {{\varvec{M}}}_{\textbf{U}} {{\varvec{J}}}{{\varvec{f}}})(\xi ))\\&= \bar{\xi }\textbf{U}(\xi ) J(({{\varvec{M}}}_{\textbf{U}} {{\varvec{J}}}{{\varvec{f}}})(\xi ))\\&= \bar{\xi }\textbf{U}(\xi ) J(({{\varvec{J}}}{{\varvec{M}}}_{\textbf{U}^{*}} {{\varvec{f}}})(\xi ))\\&= \bar{\xi }\textbf{U}(\xi ) \textbf{U}^{*}(\xi ) {{\varvec{f}}}(\xi )\\&= \bar{\xi }{{\varvec{f}}}(\xi )\\&= ({{\varvec{M}}}_{\bar{\xi }}\,{{\varvec{f}}})(\xi ). \end{aligned}$$

$$\square $$

## Conjugations and bilateral shifts

Many interesting, and naturally occurring, unitary operators are bilateral shifts. Examples include the Hilbert and Fourier transforms on $L^2(\mathbb {R})$; the translation operator $(U f)(x) = f(x - 1)$ on $L^2(\mathbb {R})$; the dilation operator $(U f)(x) = \sqrt{2} f(2 x)$ on $L^2(\mathbb {R})$; and the special class of multiplication operators $U f = \psi f$ on $L^2(m, \mathbb {T})$ (where $\psi$ is an inner function) which will be the focus of the next section. The fact that the above operators are bilateral shifts was carefully explained in [29]. This section gives an initial description of $\mathscr {C}_s(U)$ for this class of operators. Another description will be discussed in the next section. We begin with precisely what we mean by the term “bilateral shift”.

### Definition 6.1

A unitary operator *U* on $\mathcal {H}$ is a *bilateral shift* if there is a subspace $\mathcal {M} \subseteq \mathcal {H}$ for which

- $$U^{n} \mathcal {M} \perp \mathcal {M}$$ for all $$n \in \mathbb {Z}{\setminus } \{0\}$$;
- $${\displaystyle \mathcal {H}= \bigoplus _{n = -\infty }^{\infty } U^{n} \mathcal {M}}$$.

In the above, note that $U^{-1} = U^{*}$. The subspace $\mathcal {M}$ is called an *associated wandering subspace* for the bilateral shift *U*. Of course there is *the* bilateral shift $M_{\xi }$ on $L^2(m, \mathbb {T})$ defined by $(M_{\xi } f)(\xi ) = \xi f(\xi )$ where the wandering subspace $\mathcal {M}$ is the space of constant functions.

Though the wandering subspace $\mathcal {M}$ in Definition 6.1 is not unique, its dimension is [26]. The term “bilateral shift ” comes from the fact that since

$$\begin{aligned} \mathcal {H}=\bigoplus _{n=-\infty }^{\infty }\, U^n \mathcal {M}, \end{aligned}$$

every $\textbf{x} \in \mathcal {H}$ can be uniquely represented as

$$\begin{aligned} \textbf{x} = \sum _{n = -\infty }^{\infty } U^n \textbf{x}_n, \; \text{ where }\, \textbf{x}_n \in \mathcal {M}\hbox { for all }n \in \mathbb {Z}. \end{aligned}$$

This allows us to define a natural unitary operator

6.2 $$\begin{aligned} W: \mathcal {H}\rightarrow \mathscr {L}^2(m,\mathcal {M}), \quad W\left( \bigoplus _{n = -\infty }^{\infty }U^n \textbf{x}_n\right) =\sum _{n = -\infty }^{\infty } \textbf{x}_n \xi ^n. \end{aligned}$$

Moreover, $WUW^{*}={{\varvec{M}}}_\xi ,$ where ${{\varvec{M}}}_{\xi }$ is the bilateral shift from (5.2) defined on $\mathscr {L}^2(m, \mathcal {M})$ by

$$\begin{aligned} ({{\varvec{M}}}_{\xi } {{\varvec{f}}})(\xi ) = \xi {{\varvec{f}}}(\xi ), \quad {{\varvec{f}}}(\xi ) = \sum _{n = -\infty }^{\infty } \textbf{x}_{n} \xi ^n \in \mathscr {L}^2(m, \mathcal {M}). \end{aligned}$$

### Example 6.2

Examples of bilateral shifts come from a variety of places. These operators were explored in [29] using a Wold-type decomposition.

- If $U: L^2(\mathbb {R}) \rightarrow L^2(\mathbb {R})$ is the unitary translation operator defined by $(Uf)(x) = f(x - 1)$ and one sets $\mathcal {M} = \chi _{[0, 1]} L^2(\mathbb {R})$, then $\mathcal {M}$ satisfies conditions (a) and (b) of Definition 6.1 and hence *U* is unitarily equivalent to ${{\varvec{M}}}_{\xi }$ on $\mathscr {L}^2(m, \mathcal {M})$.
- Let $U:L^2(\mathbb {R}) \rightarrow L^2(\mathbb {R})$ be the unitary dilation operator defined by $(Uf)(x) = \sqrt{2} f(2x)$. If
  $$\begin{aligned} \psi (x) = {\left\{ \begin{array}{ll} 1 &{} \text{ for }\,0 \leqslant x < \frac{1}{2},\\ -1 &{} \text{ for }\,\frac{1}{2} \leqslant x \leqslant 1,\\ 0 &{} \text{ otherwise }, \end{array}\right. } \end{aligned}$$
  then (Haar) wavelet theory [6] says that the functions
  $$\begin{aligned} \psi _{n, k}(x):= 2^{\frac{n}{2}} \psi (2^{n} x - k), \quad n, k \in \mathbb {Z}, \end{aligned}$$
  form an orthonormal basis for $L^2(\mathbb {R})$. For fixed $\ell \in \mathbb {Z}$, define
  $$\begin{aligned} \mathcal {W}_{\ell }:= \bigvee \{\psi _{\ell , k}: k \in \mathbb {Z}\}. \end{aligned}$$
  Then $\mathcal {W}_{\ell } \perp \mathcal {W}_{\ell '}$ for all $\ell , \ell ' \in \mathbb {Z}, \ell \not = \ell '$, and $U \mathcal {W}_{\ell } = \mathcal {W}_{\ell + 1}$ for all $\ell \in \mathbb {Z}$. This means that the subspace $\mathcal {M} = \mathcal {W}_{0}$ satisfies conditions (a) and (b) of Definition 6.1 and hence *U* is unitarily equivalent to ${{\varvec{M}}}_{\xi }$ on $\mathscr {L}^2(m, \mathcal {M})$.
- For an inner function $\psi$ (a bounded analytic function on $\mathbb {D}$ whose radial boundary values are unimodular for *m*-almost every $\xi \in \mathbb {T}$), the operator $M_{\psi } f = \psi f$ is unitary on $L^2 = L^2(m, \mathbb {T})$ and the model space $\mathcal {M} = \mathcal {K}_{\psi } = H^2 \cap (\psi H^2)^{\perp }$ satisfies the hypothesis of conditions (a) and (b) of Definition 6.1 [29, Proposition 5.17]. Hence $M_{\psi }$ is unitarily equivalent to ${{\varvec{M}}}_{\xi }$ on $\mathscr {L}^2(m, \mathcal {M})$. Observe that if *U* is any bilateral shift on $\mathcal {H}$ with wandering subspace $\mathcal {N}$ with ${\text {dim}} \mathcal {N} = N \in \mathbb {N}\cup \{\infty \}$, then the above discussion shows that *U* is unitarily equivalent to $M_{\psi }$ on $L^2$, where $\psi$ is any inner function whose degree is *N*. When the inner function $\psi$ is a finite Blaschke product with *N* zeros (repeated according to multiplicity), the *degree of* $\psi$ is defined to be *N*. In all other cases, the degree of $\psi$ is defined to be $N = \infty$. In the next section, will give a concrete description of $\mathscr {C}_s(M_{\psi })$ written in terms of operators on $L^2$.

For a bilateral shift *U* on $\mathcal {H}$ we wish to describe $\mathscr {C}_s(U)$. Since $W U W^{*} = {{\varvec{M}}}_{\xi }$ on $\mathscr {L}^2(m, \mathcal {M})$, where $W: \mathcal {H}\rightarrow \mathscr {L}^2(m, \mathcal {M})$ is the unitary operator from (6.2), Lemma 2.3 says that $\mathfrak {C}=WCW^{*}$ is a conjugation on $\mathscr {L}^2(m,\mathcal {M})$ such that $\mathfrak {C}\textbf{M}_\xi \mathfrak {C}=\textbf{M}_{\bar{\xi }}$. The following result from [5, Thm. 4.8] describes $\mathfrak {C}$.

### Theorem 6.4

For a conjugation $\mathfrak {C}$ on $\mathscr {L}^2(m,\mathcal {M})$, the following are equivalent.

- $$\mathfrak {C}\in \mathscr {C}_{s}(\textbf{M}_\xi )$$;
- There is a $\textbf{C}\in \mathscr {L}^\infty (m, \mathscr {A}\!\mathcal {B}(\mathcal {M}))$ such that $\mathfrak {C}={{\varvec{A}}}_{{\textbf{C}}}$ and $\textbf{C}(\xi )$ is a conjugation for almost all $\xi \in \mathbb {T}$;
- For any conjugation *J* on $\mathcal {M}$ there is a $\textbf{U}\in \mathscr {L}^\infty (m,\mathcal {B}(\mathcal {M}))$ such that $\textbf{U}(\xi )$ is a *J*–symmetric unitary operator for almost all $\xi \in \mathbb {T}$ and $\mathfrak {C}= {{\varvec{M}}}_{\textbf{U}}{\textbf{J}}$.

This yields a description of $\mathscr {C}_s(U)$ when *U* is a bilateral shift. Recall the unitary operator $W: \mathcal {H}\rightarrow L^2(m, \mathcal {M})$ from (6.2) associated with a bilateral shift with wandering subspace $\mathcal {M}$.

### Corollary 6.5

Let *U* be a bilateral shift on $\mathcal {H}$ with an associated wandering subspace $\mathcal {M}$. For a conjugation *C* on $\mathcal {H}$, the following are equivalent:

- $$C \in \mathscr {C}_s(U)$$;
- $\mathfrak {C}= WC W^{*}$ is a conjugation on $\mathscr {L}^2(m, \mathcal {M})$ that satisfies any of the equivalent conditions of Theorem 6.4.

### Example 6.6

For the bilateral shift $M_{\xi }$ on $L^2 = L^2(m, \mathbb {T})$, we can examine the set $\mathscr {C}_s(M_{\xi })$. Here $\mathcal {M} = \mathbb {C}$ (the constant functions) and $J: \mathbb {C}\rightarrow \mathbb {C}$ is $J z = \overline{z}$ and so ${{\varvec{J}}}: L^2 \rightarrow L^2$ is ${{\varvec{J}}}f = \overline{f}$. If $u \in L^{\infty }$ is unimodular on $\mathbb {T}$, then $M_u f = u f$ is unitary and ${{\varvec{J}}}M_u {{\varvec{J}}}= M_{\bar{u}}$. Theorem 6.4 says that any $C \in \mathscr {C}_s(M_{\xi })$ must take the form $C = M_u {{\varvec{J}}}$ for some unimodular $u \in L^{\infty }$. We will see another path to this result in Example 7.10.

### Example 6.7

In a similar way as with the previous example, let $\mathscr {B} = \{\textbf{v}_k\}_{k \geqslant 1}$ be an orthonormal basis for $\mathcal {M}$. Here $N = {\text {dim}} \mathcal {M}$ (which might be infinite). Recall the conjugation $J_{\mathscr {B}}$ on $\mathcal {M}$ defined by $J_{\mathscr {B}} \textbf{v}_{k} = \textbf{v}_k$ for all $k \geqslant 1$ (see (2.9)). Suppose $\textbf{U}\in \mathscr {L}^{\infty }(m, \mathcal {B}(\mathcal {M}))$ is such that $\textbf{U}(\xi )$ is unitary for almost every $\xi \in \mathbb {T}$. One can check that $[J \textbf{U}(\xi ) J]_{\mathscr {B}} = \overline{[\textbf{U}(\xi )]_{\mathscr {B}}}$ for almost every $\xi \in \mathbb {T}$. Thus, for $J \textbf{U}(\xi ) J = \textbf{U}(\xi )^{*}$ it must be the case that $[\textbf{U}(\xi )^{*}]_{\mathscr {B}} = \overline{[\textbf{U}(\xi )]_{\mathscr {B}}}$. This means that every conjugation $\mathfrak {C}$ on $\mathscr {L}^2(m, \mathcal {M})$ for which $\mathfrak {C}{{\varvec{M}}}_{\xi } \mathfrak {C}= {{\varvec{M}}}_{\bar{\xi }}$ must take the form $(\mathfrak {C}{{\varvec{f}}})(\xi ) = \textbf{U}(\xi ) J({{\varvec{f}}}(\xi ))$, where $[\textbf{U}(\xi )^{*}]_{\mathscr {B}} = \overline{[\textbf{U}(\xi )]_{\mathscr {B}}}$ for almost every $\xi \in \mathbb {T}$. Note that for *any* conjugation *J* on $\mathcal {M}$, we can find an orthonormal basis $\mathscr {B}$ for the $\mathcal {M}$ for which $J = J_{\mathscr {B}}$ [18, Lemma 1]. Thus, in a way, the example above is canonical.

The results from [29, Theorem 3.3] show that for any unitary operator *U* on $\mathcal {H}$ we have $\mathcal {H}=\mathcal {K}\oplus \mathcal {K}^\prime ,$ where $\mathcal {K}$ and $\mathcal {K}'$ reducing subspaces for *U*, where $U|_{\mathcal {K}}$ is a bilateral shift and $U|_{\mathcal {K}^\prime }$ has no a bilateral shift part. For a given conjugation *C*, even assuming that *U* is *C*-symmetric, the conjugation *C* cannot be nesseserily decomposed according to this decomposition.

### Example 6.8

Consider the same operator as in [29, Example 5.6]. Namely, let $\mathcal {H}=L^2(\Omega _1)\oplus L^2(\Omega _2)\oplus L^2(\Omega _1)$, where $\Omega _1$, $\Omega _2$ are (Lebesgue) measurable disjoint subsets of $\mathbb {T}$ such that $\Omega _1 \cup \Omega _2=\mathbb {T}$ and

$$\begin{aligned} U(f,g,h)(z)=(\xi f(\xi ), \xi g(\xi ), \xi h(\xi ))\quad \text {for}\quad f,h\in L^2(\Omega _1),g\in L^2(\Omega _2). \end{aligned}$$

Define the conjugation

$$\begin{aligned} C(f,g,h)(\xi )=(\overline{h(\xi )},\overline{g(\xi )},\overline{f(\xi )})\; \; \text {for}\; \; f,h\in L^2(\Omega _1),g\in L^2(\Omega _2) \end{aligned}$$

and observe that $CUC=U^*$. As in [29, Example 5.6], the decomposition $\mathcal {H}=\mathcal {K}\oplus L^2(\Omega _1)$ with $\mathcal {K}=L^2(\mathbb {T})=L^2(\Omega _1)\oplus L^2(\Omega _2)$, is a desired bilateral shift decomposition, but it is not *C* invariant ($C\mathcal {K}\nsubseteq \mathcal {K}$).

## Unitary multiplication operators on $L^2(m, \mathbb {T})$

As mentioned in Example 6.2(c), we have a model for any bilateral shift *U* on $\mathcal {H}$ as the multiplication operator $M_{\psi }$ on $L^2 = L^2(m, \mathbb {T})$, where $\psi$ is an inner function whose degree is that of the (uniquely defined) dimension of any wandering subspace for *U*. In this section we give a concrete and tangible description of $\mathscr {C}_s(M_{\psi })$. If *J* is the conjugation $J f = \overline{f}$ on $L^2$ and $C \in \mathscr {C}_s(M_{\psi })$, then $A = C J$ is a unitary operator on $L^2$ for which $A M_{\psi } = M_{\psi } A$. Thus, in order to describe $\mathscr {C}_{s}(M_{\psi })$, we first need to characterize the bounded operators on $L^2$ that commute with $M_{\psi }$.

### Remark 7.1

For an inner function $\psi$, a known result (see for example [29, Proposition 5.17]), says that

7.2 $$\begin{aligned} L^2 = \bigoplus _{n = -\infty }^{\infty } \psi ^{n} \mathcal {K}_{\psi }, \end{aligned}$$

where $\mathcal {K}_{\psi } = H^2 \ominus \psi H^2$ is the model space associated with $\psi$. In other words, $\mathcal {K}_{\psi }$ is a wandering subspace for $M_{\psi }$ (recall Definition 6.1). Let us set up some notation to be used below. Let $N = {\text {dim}} \mathcal {K}_{\psi } \in \mathbb {N}\cup \{\infty \}$. Observe that *N* is finite if and only if $\psi$ is a finite Blaschke product with *N* zeros, repeated according to multiplicity [15, Prop. 5.19]. Also define the space

$$\begin{aligned} \bigoplus _{1 \leqslant j \leqslant N} L^2 = L^2 \oplus L^2 \oplus \cdots \oplus L^2. \end{aligned}$$

The norm of an element ${{\varvec{f}}}= [f_{j}]_{1 \leqslant j \leqslant N}^{t} \in \bigoplus _{1 \leqslant j \leqslant N} L^2$ is

$$\begin{aligned} \Vert {{\varvec{f}}}\Vert = \left( \sum _{1 \leqslant j \leqslant N} \Vert f_j\Vert ^{2}_{L^2}\right) ^{1/2}. \end{aligned}$$

When $N = \infty$, we need to assume that the sum defining $\Vert {{\varvec{f}}}\Vert$ is finite. Furthermore, the operator $\bigoplus _{1 \leqslant j \leqslant N} M_{\xi }$ (called the *inflation* of the bilateral shift $M_{\xi }$ on $L^2$) is given by

$$\begin{aligned} \left( \bigoplus _{1 \leqslant j \leqslant N} M_{\xi }\right) {{\varvec{f}}}(\xi ) = \xi {{\varvec{f}}}(\xi ) = [\xi f_{j}(\xi )]^{t}_{1 \leqslant j \leqslant N}. \end{aligned}$$

We also define

$$\begin{aligned} \ell ^{2}_{N}:= \left\{ \textbf{x} = [x_j]^{t}_{1 \leqslant j \leqslant N}, x_j \in \mathbb {C}: \Vert \textbf{x}\Vert _{\ell ^{2}_{N}} = \left( \sum _{1 \leqslant j \leqslant N} |x_j|^2\right) ^{1/2} < \infty \right\} . \end{aligned}$$

When $N = \infty$, note that $\ell ^{2}_{N}$ is equal to $\ell ^{2}_{+}$ which was the space discussed earlier in (2.8). Finally, observe that

7.3 $$\begin{aligned} \bigoplus _{1 \leqslant j \leqslant N} M_{\xi } \cong {{\varvec{M}}}_{\xi }|_{\mathscr {L}^2(m, \ell ^{2}_{N})}. \end{aligned}$$

### Theorem 7.4

For an inner function $\psi$, let $\{h_j\}_{1 \leqslant j \leqslant N}$ be an orthonormal basis for the model space $\mathcal {K}_{\psi }$. Then we have the following.

- Every $f \in L^2$ has the unique decomposition
  7.5 $$\begin{aligned} f = \sum _{1 \leqslant j \leqslant N} h_j \cdot (f_j \circ \psi ), \end{aligned}$$
  where each $f_j$ belongs to $L^2$ and $\Vert f\Vert = (\sum _{1 \leqslant j \leqslant N} \Vert f_j\Vert ^2)^{\frac{1}{2}}$.
- The operator
  $$\begin{aligned} W: L^2 \rightarrow \bigoplus _{1 \leqslant j \leqslant N} L^2, \quad W f = [f_1 \; f_2\; f_3\; \ldots ]^{t}, \end{aligned}$$
  is unitary and $W^{*} [k_j]^{t}_{1 \leqslant j \leqslant N} = \sum _{1 \leqslant j \leqslant N}h_j \cdot (k_j \circ \psi ).$
- $$W M_{\psi } W^{*} = \bigoplus _{1 \leqslant j \leqslant N} M_{\xi }$$ and $$W M_{\overline{\psi }} W^{*} = \bigoplus _{1 \leqslant j \leqslant N} M_{\bar{\xi }}$$.
- If $A \in \mathcal {B}(L^2)$ satisfies $A M_{\psi } = M_{\psi } A$, then
  $$\begin{aligned} A f = \sum _{1 \leqslant j \leqslant N} (f_j \circ \psi ) \sum _{1 \leqslant k \leqslant N} h_k \cdot (\varphi _{k j} \circ \psi ), \end{aligned}$$
  where $$\Phi = [\varphi _{i j}]_{1 \leqslant i, j \leqslant N}\in \mathscr {L}^{\infty }(m,\mathcal {B}(\ell ^2_N))$$.
- If $\Phi = [\varphi _{i j}]_{1 \leqslant i, j \leqslant N}\in \mathscr {L}^{\infty }(m,\mathcal {B}(\ell ^2_N))$, then the operator *A* defined in (d) is unitarily equivalent to $M_{\Phi }$ on $\bigoplus _{1 \leqslant j \leqslant N} L^2$, i.e.,
  $$\begin{aligned}{}[f_j]^{t}_{1 \leqslant j \leqslant N} \mapsto \Phi [f_j]^{t}_{1 \leqslant j \leqslant N}. \end{aligned}$$

### Proof

By (7.2), every $f \in L^2$ can be written (uniquely) as

7.6 $$\begin{aligned} f = \sum _{j = -\infty }^{\infty } \psi ^{j} \sum _{1 \leqslant k \leqslant N} a_{j k} h_k. \end{aligned}$$

Moreover, since $\psi ^j K_{\psi } \perp \psi ^{j'} K_{\psi }$ for all $j \not = j'$ and $\{h_k\}_{1 \leqslant k \leqslant N}$ forms an orthonormal basis for $\mathcal {K}_{\psi }$, we have

7.7 $$\begin{aligned} \Vert f\Vert ^2 = \sum _{j = -\infty }^{\infty } \sum _{1 \leqslant k \leqslant N} |a_{jk}|^2 < \infty . \end{aligned}$$

Rewrite the expression in (7.6) as

$$\begin{aligned} f = \sum _{1 \leqslant k \leqslant N} h_k \sum _{j = -\infty }^{\infty } a_{jk} \psi ^{j}. \end{aligned}$$

By (7.7) the function $f_k = \sum _{j = -\infty }^{\infty } a_{jk} z^{j}$ belongs to $L^2$ and thus,

$$\begin{aligned} f = \sum _{1 \leqslant k \leqslant N} h_k \cdot (f_k \circ \psi ). \end{aligned}$$

Finally, by Parseval’s theorem and (7.7), note that

$$\begin{aligned} \sum _{1 \leqslant k \leqslant N} \Vert f_k\Vert ^2&= \sum _{1 \leqslant k \leqslant N} \sum _{j = -\infty }^{\infty } |a_{jk}|^2 = \Vert f\Vert ^2. \end{aligned}$$

This verifies statements (a) and (b). To verify statement (c), note that

$$\begin{aligned} W M_{\psi } W^{*} [k_j]^{t}_{1 \leqslant j \leqslant N}&= W M_{\psi } \sum _{1 \leqslant j \leqslant N} h_j \cdot (k_j \circ \psi )\\&= W \sum _{1 \leqslant j \leqslant N} h_j \cdot \psi \cdot (k_j \circ \psi )\\&= W \sum _{1 \leqslant j \leqslant N} h_j \cdot ((\xi k_j) \circ \psi )\\&= [\xi k_j(\xi )]^{t}_{1 \leqslant j \leqslant N}. \end{aligned}$$

This shows that $W M_{\psi } W^{*} = \bigoplus _{1 \leqslant j \leqslant N} M_{\xi }$. Similarly, one can verify the second equality in (c).

To prove (d) and (e), recall from [33, Chapter III] that the bounded operators on the space $\bigoplus _{1 \leqslant j \leqslant N} L^2$ that commute with $\bigoplus _{1 \leqslant j \leqslant N} M_{\xi }$ must take the form of multiplication by the matrix function $\Phi = [\varphi _{ij}]_{1 \leqslant i, j \leqslant N} \in \mathscr {L}^{\infty }(m, \ell ^2_N)$. Putting this all together, we see that if $A \in \mathcal {B}(L^2)$ commutes with $M_{\psi }$, then $W A W^{*}$ commutes with $\bigoplus _{1 \leqslant j \leqslant N} M_{\xi }$ and so $W A W^{*} = {{\varvec{M}}}_{\Phi },$ equivalently $A = W^{*} {{\varvec{M}}}_{\Phi } W$. This translates to an operator on $L^2$ by

$$\begin{aligned} A f&= W^{*} \Phi W f\\&= W^{*} [\varphi _{ij} ]_{1 \leqslant i, j \leqslant N}[f_1 \; f_2 \; \ldots ]^{t}\\&= W^{*} \left[ \sum _{1 \leqslant j \leqslant N} \varphi _{1j} f_j \; \; \sum _{1 \leqslant j \leqslant N} \varphi _{2j} f_j \; \; \sum _{1 \leqslant j \leqslant N} \varphi _{3j} f_j \; \; \ldots \right] ^{t}\\&= h_1\cdot \left( \sum _{1 \leqslant j \leqslant N} \varphi _{1j} f_j\right) \circ \psi + h_2 \cdot \left( \sum _{1 \leqslant j \leqslant N} \varphi _{2j} f_j\right) \circ \psi + \ldots \\&= (f_{1} \circ \psi ) \cdot \left( \sum _{1 \leqslant k \leqslant N} h_{k} \cdot (\varphi _{k1} \circ \psi )\right) + (f_{2} \circ \psi ) \cdot \left( \sum _{1 \leqslant k \leqslant N} h_{k} \cdot (\varphi _{k2} \circ \psi )\right) + \cdots , \end{aligned}$$

which completes the proof of (d) and (e).

From our earlier discussion, we know that the standard conjugation $J f = \bar{f}$ on $L^2$ induces the standard conjugation on ${{\varvec{J}}}$ on $\bigoplus _{1 \leqslant j \leqslant N} L^{2}$ by

$$\begin{aligned} {{{\varvec{J}}}}\textbf{F}= [ \bar{f}_1 \; \bar{f}_2 \; \ldots ]^{t}=\overline{\textbf{F}} \end{aligned}$$

Here is our description of $\mathscr {C}_{s}(M_{\psi })$ when $\psi$ is inner.

### Theorem 7.8

Suppose that $\psi$ is an inner function and $\{h_j\}_{1 \leqslant j \leqslant N}$ is an orthonormal basis for $\mathcal {K}_{\psi }$. Then $C\in \mathscr {C}_s(M_\psi )$ if and only if there is a $\Phi = [\varphi _{i j}]_{1 \leqslant i, j \leqslant N} \in \mathscr {L}^{\infty }(m, \ell ^{2}_{N})$ such that

- $\Phi ^{*} \Phi = I$ almost everywhere on $\mathbb {T}$;
- $\Phi ^{t} = \Phi$ almost everywhere on $\mathbb {T}$;
- ${\displaystyle C f = \sum \nolimits _{1 \leqslant j \leqslant N} (\bar{f}_j \circ \psi ) \sum \nolimits _{1 \leqslant k \leqslant N} h_k \cdot (\varphi _{k, j} \circ \psi ),}$ for all $f \in L^2$ with the decomposition from (7.5).

### Proof

Suppose $C \in \mathscr {C}_{s}(M_{\psi })$. Note that $\widetilde{C}:=WCW^*$ defines a conjugation on $\bigoplus _{1 \leqslant j \leqslant N} L^2$. Since $C M_{\psi } C = M_{\overline{\psi }}$, we can use Theorem 7.4(c) to see that

$$\begin{aligned} \widetilde{C}\left( \bigoplus _{1 \leqslant j \leqslant N}M_{\xi } \right) \widetilde{C} = \bigoplus _{1 \leqslant j \leqslant N}M_{\bar{\xi }}. \end{aligned}$$

Since $\bigoplus _{1 \leqslant j \leqslant N}M_{\xi }$ on $\bigoplus _{1 \leqslant j \leqslant N}L^2$ is unitary equivalent to ${{\varvec{M}}}_{\xi }$ on $\mathscr {L}^2(m,\ell ^2_{N})$ (recall (7.3)), Theorem 6.4 yields (with the choice of conjugation *J* on $\ell ^2_{N}$ being $J \textbf{x} = \overline{\textbf{x}}$), a matrix-valued function $\Phi = [\varphi _{i j}]_{1 \leqslant i, j \leqslant N} \in \mathscr {L}^{\infty }(m, \ell ^{2}_{N})$ that is unitary valued *m*-almost everywhere, i.e., $\Phi \Phi ^{*} = \Phi ^{*} \Phi = I$, and such that $\widetilde{C}={{\varvec{M}}}_\Phi {{\varvec{J}}}$. Moreover, since $\widetilde{C}^2=I$ we see that

$$\begin{aligned} I = ({{\varvec{M}}}_{\Phi } {{\varvec{J}}})({{\varvec{M}}}_{\Phi } {{\varvec{J}}}) = {{\varvec{M}}}_{\Phi } ({{\varvec{J}}}{{\varvec{M}}}_{\Phi } {{\varvec{J}}}) = {{\varvec{M}}}_{\Phi } {{\varvec{M}}}_{\overline{\Phi }} \end{aligned}$$

and so $\overline{\Phi } = \Phi ^{*}$. Combine this with the previous identity to see that $\Phi ^{t} = \Phi$. So far we have shown that if $C \in \mathscr {C}_{s}(M_{\psi })$ then $C = W^{*} ({{\varvec{M}}}_{\Phi } {{\varvec{J}}})W$ where $\Phi$ satisfies the conditions of (a) and (b). If $\Phi$ satisfies the conditions (a) and (b) then a short argument will show that $\Phi$ is unitary valued almost everywhere and the condition (b) will show that $J \Phi J = \Phi ^{*}$ almost everywhere. Proposition 5.6 now says that ${{\varvec{M}}}_{\Phi } {{\varvec{J}}}\in \mathscr {C}_{s}({{\varvec{M}}}_{\xi })$ and hence $W^{*} ({{\varvec{M}}}_{\Phi } {{\varvec{J}}}) W \in \mathscr {C}_{s}(M_{\psi })$. So we have shown that $C \in \mathscr {C}_{s}(M_{\psi })$ if and only if $C = W^{*} ({{\varvec{M}}}_{\Phi } {{\varvec{J}}}) W$, where $\Phi$ satisfies the conditions in (a) and (b).

It remains to verify the formula in (c). Observe that for all $f = \sum _{1 \leqslant j \leqslant N} h_j \cdot (f_j \circ \psi )\in L^2$,

$$\begin{aligned} C f&= W^{*} {{\varvec{M}}}_\Phi {{\varvec{J}}}W f\\&= W^{*} [\varphi _{ij} ]_{1 \leqslant i, j \leqslant N}[\bar{f}_1 \; \bar{f}_2 \; \ldots ]^{t}\\&= W^{*} \left[ \sum _{1 \leqslant j \leqslant N} \varphi _{1j} \bar{f}_j \; \; \sum _{1 \leqslant j \leqslant N} \varphi _{2j} \bar{f}_j \; \; \sum _{1 \leqslant j \leqslant N} \varphi _{3j} \bar{f}_j \; \; \ldots \right] ^{t}\\&= h_1\cdot \left( \sum _{1 \leqslant j \leqslant N} \varphi _{1j} \bar{f}_j\right) \circ \psi + h_2 \cdot \left( \sum _{1 \leqslant j \leqslant N} \varphi _{2j} \bar{f}_j\right) \circ \psi + \ldots \\&= (\bar{f}_{1} \circ \psi ) \cdot \left( \sum _{1 \leqslant k \leqslant N} h_{k} \cdot (\varphi _{k1} \circ \psi )\right) + (\bar{f}_{2} \circ \psi ) \cdot \left( \sum _{1 \leqslant k \leqslant N} h_{k} \cdot (\varphi _{k2} \circ \psi )\right) + \cdots . \end{aligned}$$

Conversely, suppose there is a $\Phi = [\varphi _{i j}]_{1 \leqslant i, j \leqslant N} \in \mathscr {L}^{\infty }(m, \ell ^{2}_{N})$ such that conditions (a), (b), and (c) hold. Theorem 7.4(e) and the argument above shows that $C \in \mathscr {C}_{s}(M_{\psi })$.

### Remark 7.9

The description of $\mathscr {C}_{s}(M_{\psi })$ for an inner function $\psi$ depends on knowing an orthonormal basis for the model space $\mathcal {K}_{\psi }$. There are several “natural” bases one could choose. See [15, Prop. 5.25] for some particular examples when ${\text {dim}} \mathcal {K}_{\psi }$ is finite.

### Example 7.10

Suppose $\psi (z) = z$. In this case, $M_{\psi }$ becomes *the* bilateral shift $M_{\xi }$ on $L^2$. Furthermore, $\mathcal {K}_{\psi } = \mathbb {C}$ (the constant functions) and the expansion of an $f \in L^2$ from Theorem 7.4 becomes the classical Fourier expansion. Finally, Theorem 7.4 says that every $C \in \mathscr {C}_{s}(M_{\xi })$ must take the form $(C f)(\xi ) = u(\xi ) \overline{f(\xi )}$ for some $u \in L^{\infty }$ that is unimodular almost everywhere. We observed this by a different method in Example 6.6.

### Example 7.11

When $\psi (z) = z^2$ above, one can check that the functions $h_{1}(z) \equiv 1$ and $h_{2}(z) = z$ form an orthonormal basis for $\mathcal {K}_{\psi }$. Furthermore, using the notation from Theorem 7.4,

7.12 $$\begin{aligned} f_1(\xi ) = \sum _{j = -\infty }^{\infty } \widehat{f}(2 j) \xi ^j \quad \text{ and } \quad f_{2}(\xi ) = \sum _{j = -\infty }^{\infty } \widehat{f}(2 j + 1) \xi ^j. \end{aligned}$$

Then, one can check that

$$\begin{aligned} f(\xi ) = h_{1}(\xi ) f_{1}(\xi ^2) + h_{2}(\xi ) f_{2}(\xi ^2) = f_{1}(\xi ^2) + \xi f_{2}(\xi ^2). \end{aligned}$$

Theorem 7.8 says that every $C \in \mathscr {C}_s(M_{\xi ^2})$ takes the form

$$\begin{aligned} (C f)(\xi ) = \overline{ f_{1}(\xi ^2)} (\varphi _{11}(\xi ^2) + \xi \varphi _{21}(\xi ^2)) + \overline{ f_{2}(\xi ^2)} (\varphi _{21}(\xi ^2) + \xi \varphi _{22}(\xi ^2)), \end{aligned}$$

where $\varphi _{ij}$, $1 \leqslant i, j \leqslant 2$, are bounded measurable functions on $\mathbb {T}$ for which $\varphi _{12}(\xi ) = \varphi _{21}(\xi )$ and

7.13 $$\begin{aligned} \begin{bmatrix} \overline{ \varphi _{11}(\xi )} &{} \overline{ \varphi _{21}(\xi )}\\ \overline{ \varphi _{21}(\xi )} &{} \overline{ \varphi _{22}(\xi )} \end{bmatrix} \begin{bmatrix} \varphi _{11}(\xi ) &{} \varphi _{21}(\xi )\\ \varphi _{21}(\xi ) &{} \varphi _{22}(\xi ) \end{bmatrix}= \begin{bmatrix} 1 &{} 0\\ 0 &{} 1 \end{bmatrix} \end{aligned}$$

for almost every $\xi \in \mathbb {T}$. The condition from (7.13) is equivalent to the identities

$$\begin{aligned}{} & {} |\varphi _{11}(\xi )|^2+|\varphi _{21}(\xi )|^2= 1,\\{} & {} |\varphi _{11}(\xi )|=|\varphi _{22}(\xi )|,\\{} & {} \overline{ \varphi _{11}(\xi )}\varphi _{21}(\xi )+\overline{\varphi _{21}(\xi )}\varphi _{22}(\xi )=0 \end{aligned}$$

for almost every $\xi \in \mathbb {T}$.

To make this more tangible, let $t={\text {Arg}}(\xi )\in (-\pi ,\pi ]$ and let $s(t),\alpha (t)$, $\beta (t)$, and $\gamma (t)$ be any $2\pi$–periodic bounded real-valued (Lebesgue) measurable functions. Set

$$\begin{aligned} \varphi _{11}(\xi )= & {} e^{i\alpha (t)}s(t),\\ \varphi _{22}(\xi )= & {} e^{i\beta (t)}s(t),\\ \varphi _{21}(\xi )= & {} \varphi _{12}(\xi )=e^{i\gamma (t)}\sqrt{1-s^2(t)}, \end{aligned}$$

and observe that the conditions above yield

$$\begin{aligned} \gamma (t)=\tfrac{1}{2}(\pi +\alpha (t)+\beta (t)), \end{aligned}$$

which shows that

$$\begin{aligned}{} & {} 0 \leqslant s(t) \leqslant 1,\\{} & {} \varphi _{11}(\xi )=e^{i\alpha (t)}s(t),\\{} & {} \varphi _{22}(\xi )=e^{i\beta (t)}s(t),\\{} & {} \varphi _{21}(\xi ) = \varphi _{12}(\xi ) =ie^{\tfrac{i}{2}(\alpha (t)+\beta (t))}\sqrt{1-s^2(t)}. \end{aligned}$$

Using the notation from (7.12), this means that every $C \in \mathscr {C}_s(M_{\xi ^2})$ must take the form

$$\begin{aligned} (C f)(\xi )= & {} \overline{ f_{1}(\xi ^2)}\Big ( e^{i\alpha (2t)}s(2t) + i\xi e^{\tfrac{i}{2}(\alpha (2t)+\beta (2t))}\sqrt{1-s^2(2t)}\Big )\\{} & {} + \overline{ f_{2}(\xi ^2)}\Big ( i\,e^{\tfrac{i}{2}(\alpha (2t)+\beta (2t))}\sqrt{1-s^2(2t)} + \xi e^{i\beta (2t)}s(2t)\Big ), \end{aligned}$$

where $t={\text {Arg}}(\xi )\in (-\pi ,\pi ]$ and $s(t),\alpha (t),\beta (t)$ are any $2\pi$–periodic bounded real-valued measurable functions.

As a specific nontrivial example we can have the interesting $C \in \mathscr {C}_{s}(M_{\xi ^2})$ defined by

$$\begin{aligned} (C f)(\xi ) = \overline{ f_{1}(\xi ^2)} \big (\sin (2t) + \xi \cos (2t)\big ) + \overline{ f_{2}(\xi ^2)}\big (\cos (2t) - \xi \sin (2t)\big ), \end{aligned}$$

where $$t={\text {Arg}}(\xi )$$ ($$s(t) = \sin t$$, $$\alpha (t) \equiv 0$$, $$\beta (t) \equiv -\pi $$)

Another interesting example comes from setting $s(t) \equiv s$, $\alpha (t) = \lambda t$, $\beta (t) = -\pi - \lambda t$, $\lambda \in \mathbb {R}$, which yields

$$\begin{aligned} (C f)(\xi ) = \overline{f_1(\xi ^2)} ( s e^{i \lambda t} + \xi \sqrt{1 - s^2} ) + \overline{f_{2}(\xi )} (\sqrt{1 - s^2} + i \xi s e^{-i \lambda t}). \end{aligned}$$

## Conjugations via the spectral theorem

This section describes $\mathscr {C}_s(U)$ using the multiplicity version of the spectral theorem. Important applications of this are Theorem 8.6 and Theorem 8.8 below which connect the invariant subspaces of $C \in \mathscr {C}_{s}(U)$ with the hyperinvariant subspaces of *U*.

We begin with the following (multiplicity) version of the spectral theorem for unitary operators from [7, Ch. IX, Theorem 10.20]. The reader might need a refresher of the notation from §5.

### Theorem 8.1

(Spectral Theorem) For a unitary operator *U* on a separable Hilbert space $\mathcal {H}$, there are mutually singular measures $\mu _{\infty }, \mu _1, \mu _2, \ldots \in M_{+}(\mathbb {T})$, along with Hilbert spaces $\mathcal {H}_{\infty }, \mathcal {H}_{1}, \mathcal {H}_2, \ldots$ each with corresponding ${\text {dim}} \mathcal {H}_{k} = k$, $k = \infty , 1, 2, 3, \dots$, and a unitary operator

$$\begin{aligned} \mathcal {I}: \mathcal {H}\rightarrow \mathscr {L}^2(\mu _\infty ,\mathcal {H}_\infty )\oplus \mathscr {L}^2(\mu _1,\mathcal {H}_1)\oplus \mathscr {L}^2(\mu _2,\mathcal {H}_2)\oplus \cdots \end{aligned}$$

such that $\mathcal {I}U \mathcal {I}^{*}$ is equal to the unitary operator

$$\begin{aligned} {{\varvec{M}}}_{\xi }^{(\infty )} \oplus {{\varvec{M}}}_{\xi }^{(1)} \oplus {{\varvec{M}}}_{\xi }^{(2)} \oplus \cdots , \end{aligned}$$

where for $$i = \infty , 1, 2, 3, \ldots $$,

$$\begin{aligned} {{\varvec{M}}}_{\xi }^{(i)}: \mathscr {L}^2(\mu _{i}, \mathcal {H}_{i}) \rightarrow \mathscr {L}^2(\mu _{i}, \mathcal {H}_{i}), \quad {{\varvec{M}}}_{\xi }^{(i)} {{\varvec{f}}}(\xi ) = \xi {{\varvec{f}}}(\xi ). \end{aligned}$$

The main driver of the results of this section is the following.

### Theorem 8.2

Let *U* be a unitary operator on a separable Hilbert space $\mathcal {H}$ with spectral representation as in Theorem 8.1. For a conjugation *C* on $\mathcal {H}$, the following are equivalent.

- $$C \in \mathscr {C}_s(U)$$,
- For each $j=\infty ,1,2,\dots$ there are conjugations $\mathfrak {C}_{j}$ on $\mathscr {L}^{2}(\mu _j,\mathcal {H}_j)$ such that $\mathfrak {C}_j \in \mathscr {C}_{s}({{\varvec{M}}}_{\xi }^{(i)})$ and
  $$\begin{aligned} C={\mathcal {I}}^{*}\Big (\bigoplus \mathfrak {C}_{j}\Big )\mathcal {I}. \end{aligned}$$
- For each $j=\infty ,1,2,\dots$ there are $\textbf{C}_j \in \mathscr {L}^{\infty }(\mu _j, \mathscr {A}\!\mathcal {B}(\mathcal {H}_j))$ such that $\textbf{C}_{j}(\xi )$ is a conjugation for $\mu _{j}$ almost every $\xi \in \mathbb {T}$ and
  $$\begin{aligned} C={\mathcal {I}}^{*}\Big (\bigoplus {{\varvec{A}}}_{\textbf{C}_j}\Big )\mathcal {I}. \end{aligned}$$
- For each $i=\infty ,1,2,\dots$ and for any conjugation $J_i$ on $\mathcal {H}_i$ there is a $\textbf{U}_i \in \mathscr {L}^{\infty }(\mu _i, \mathcal {B}(\mathcal {H}_i))$ such that $\textbf{U}_i(\xi )$ is unitary and $J_i$–symmetric for $\mu _i$-almost every $\xi \in \mathbb {T}$ and
  $$\begin{aligned} C={\mathcal {I}}^{*}\Big (\bigoplus \textbf{U}_i {{\varvec{J}}}_i \Big )\mathcal {I}={\mathcal {I}}^{*}\Big (\bigoplus \textbf{U}_i \Big )\Big (\bigoplus {{\varvec{J}}}_i \Big )\mathcal {I}. \end{aligned}$$

### Proof

$$(a) \Longrightarrow (d)$$: Set

8.3 $$\begin{aligned} \widetilde{{{\varvec{M}}}}_{\xi }:= \mathcal {I}U\mathcal {I}^{*} = {{\varvec{M}}}_{ \xi }^{(\infty )} \oplus {{\varvec{M}}}_{ \xi }^{(1)} \oplus {{\varvec{M}}}_{ \xi }^{(2)} \oplus \cdots , \end{aligned}$$

and, for a conjugation *C* on $\mathcal {H}$ define $\widetilde{\!\textbf{C}}=\mathcal {I}C\mathcal {I}^{*}$ which, by Lemma 2.3, will be a conjugation on

$$\begin{aligned} \textbf{L}^2_{\mathcal {H}}:= \mathscr {L}^2(\mu _\infty ,\mathcal {H}_\infty )\oplus \mathscr {L}^2(\mu _1,\mathcal {H}_1)\oplus \mathscr {L}^2(\mu _2,\mathcal {H}_2)\oplus \cdots . \end{aligned}$$

Assuming that $C U C = U^{*}$, we see that $\widetilde{\!\textbf{C}}\,\widetilde{{{\varvec{M}}}}_\xi \,\widetilde{\!\textbf{C}}=\widetilde{{{\varvec{M}}}}_{\bar{\xi }}.$

Let $J_i$ be any a conjugation on $\mathcal {H}_i$. Then, as in (5.3), this induces a conjugation ${{\varvec{J}}}_i$ on $\mathscr {L}^2(\mu _i,\mathcal {H}_i)$ defined by

$$\begin{aligned} ({{\varvec{J}}}_i{{\varvec{f}}}_i)(\xi )=J_i({{\varvec{f}}}_i(\xi )), \; \; {{\varvec{f}}}_i\in \mathscr {L}^2(\mu _i,\mathcal {H}_i). \end{aligned}$$

Define a conjugation $\widetilde{\!{{\varvec{J}}}}$ on ${{\varvec{L}}}^2_{\mathcal {H}}$ by

$$\begin{aligned} \widetilde{\!{{\varvec{J}}}}:={{\varvec{J}}}_{\infty } \oplus {{\varvec{J}}}_1\oplus {{\varvec{J}}}_2\cdots . \end{aligned}$$

From (5.3) we see that $\widetilde{\!{{\varvec{J}}}}\,\widetilde{{{\varvec{M}}}}_\xi \,\widetilde{\!{{\varvec{J}}}}=\widetilde{{{\varvec{M}}}}_{\bar{\xi }}.$ Now observe that

$$\begin{aligned} \widetilde{{{\varvec{M}}}}_\xi (\widetilde{\!\textbf{C}}\,\widetilde{\!{{\varvec{J}}}})=(\widetilde{\!\textbf{C}}\, \widetilde{{{\varvec{M}}}}_{\bar{\xi }})\, \widetilde{\!{{\varvec{J}}}}=(\widetilde{\!\textbf{C}}\ \widetilde{\!{{\varvec{J}}}}) \widetilde{{{\varvec{M}}}}_\xi . \end{aligned}$$

This says that the operator $\widetilde{\!\textbf{C}}\ \widetilde{\!{{\varvec{J}}}}$ commutes with $\widetilde{{{\varvec{M}}}_{\xi }}$. The spectral theorem applied to $\widetilde{{{\varvec{M}}}_{\xi }}$ also yields the commutant [7, p.  307, Theorem 10.20], namely there are operator valued functions $\textbf{U}_i\in \mathscr {L}^{\infty }(\mu _i,\mathcal {B}(\mathcal {H}_i))$ for $i=\infty ,1,2,\dots$ such that

$$\begin{aligned} \widetilde{\!\textbf{C}}\ \widetilde{\!{{\varvec{J}}}}={{\varvec{M}}}_{\textbf{U}_{\infty }}\oplus {{{\varvec{M}}}}_{\textbf{U}_1}\oplus {{{\varvec{M}}}}_{\textbf{U}_{2}} \oplus \cdots . \end{aligned}$$

Since $\widetilde{\!\textbf{C}}\ \widetilde{\!{{\varvec{J}}}}$ is unitary (being linear, isometric, and onto), it follows that each $\widetilde{{{\varvec{M}}}}_{\textbf{U}_{i}}$ is unitary and, consequently, $\textbf{U}_{i}(\xi )$ is unitary for $\mu _i$-almost every $\xi \in \mathbb {T}$. Therefore,

$$\begin{aligned} \widetilde{\!\textbf{C}}=\Big (\bigoplus {{\varvec{M}}}_{\textbf{U}_i}\Big )\Big (\bigoplus {{\varvec{J}}}_{i}\Big )=\bigoplus ({{\varvec{M}}}_{\textbf{U}_i}{{\varvec{J}}}_i). \end{aligned}$$

Since each $\widetilde{\!\textbf{C}}|_{\mathscr {L}^2(\mu _i,\mathcal {H}_i)}$ is a conjugation, Proposition 5.6 says that the operator $\textbf{U}_{i}(\xi )$ is $J_{i}$–symmetric $\mu _i$ almost everywhere. Thus, we have verified the implication $(a) \Longrightarrow (d)$.

$(d) \Longrightarrow (c)$: For each $i = \infty , 1, 2, \ldots$, take $\textbf{C}_{i}=\textbf{U}_{i}{{\varvec{J}}}_{i}$.

$(c) \implies (b)$: For each $i = \infty , 1, 2, \ldots$, it is enough to take $\mathfrak {C}_i={{\varvec{A}}}_{\textbf{C}_i}$ and prove that

$$\begin{aligned} {{\varvec{A}}}_{\textbf{C}_{i}}{{\varvec{M}}}^{(i)}_{\xi }={{\varvec{M}}}^{(i)}_{\bar{\xi }}{{\varvec{A}}}_{\textbf{C}_{i}}. \end{aligned}$$

Indeed, for each ${{\varvec{f}}}_{i} \in \mathscr {L}^2(\mu _i, \mathcal {H}_i)$ we have

$$\begin{aligned} ({{\varvec{A}}}_{\textbf{C}_{i}}{{\varvec{M}}}^{(i)}_\xi {{\varvec{f}}}_i)(\xi )&=\textbf{C}_{i}(\xi )({{\varvec{M}}}^{(i)}_{\xi }{{\varvec{f}}}_i)(\xi )\\&=\textbf{C}_{i}(\xi )(\xi {{\varvec{f}}}_i(\xi ))\\&=\bar{\xi }\textbf{C}_{i}(\xi ){{\varvec{f}}}_i(\xi )\\&= \bar{\xi }({{\varvec{A}}}_{\textbf{C}_i} {{\varvec{f}}}_i)(\xi )\\&=({{\varvec{M}}}^{(i)}_{\bar{\xi }}{{\varvec{A}}}_{\textbf{C}_{i}}{{\varvec{f}}}_i)(\xi ). \end{aligned}$$

$(b) \Longrightarrow (a)$: Recalling the notation from (8.3), note that

$$\begin{aligned} CUC&=\mathcal {I}\Big (\bigoplus \mathfrak {C}_{i}\Big )\Big (\bigoplus {{\varvec{M}}}^{(i)}_\xi \Big )\Big (\bigoplus \mathfrak {C}_{i}\Big )\mathcal {I}^{*}\\&=\mathcal {I}\Big (\bigoplus \mathfrak {C}_{\,i} {{\varvec{M}}}^{(i)}_\xi \mathfrak {C}_{i}\Big )\mathcal {I}^{*}\\&=\mathcal {I}\Big (\bigoplus {{\varvec{M}}}^{(i)}_{\bar{\xi }}\Big )\mathcal {I}^{*}\\&=\mathcal {I}\widetilde{{{\varvec{M}}}_{\bar{\xi }}}\mathcal {I}^{*} =U^*. \end{aligned}$$

$$\square $$

The multiplication operator ${{\varvec{M}}}_\xi$ on $\mathscr {L}^2(\mu ,\mathcal {H})$ is a special case of Theorem 8.2 – which we record here for what follows.

### Theorem 8.4

Let $\mu \in M_{+}(\mathbb {T})$ and $\mathfrak {C}$ be a conjugation on $\mathscr {L}^2(\mu , \mathcal {H})$. Then following are equivalent.

- $$\mathfrak {C}\in \mathscr {C}_s({{\varvec{M}}}_{\xi })$$.
- There are $\textbf{C}\in \mathscr {L}^{\infty }(\mu , \mathscr {A}\!\mathcal {B}(\mathcal {H}))$ such that $\textbf{C}(\xi )$ is a conjugation for $\mu$ almost every $\xi \in \mathbb {T}$ and $\mathfrak {C}= {{\varvec{A}}}_{\textbf{C}}$.
- For any conjugation *J* on $\mathcal {H}$ there is a $\textbf{U}\in \mathscr {L}^{\infty }(\mu , \mathcal {B}(\mathcal {H}))$ such that $\textbf{U}(\xi )$ is unitary and *J*–symmetric for $\mu$ almost every $\xi \in \mathbb {T}$ and $\mathfrak {C}={{\varvec{M}}}_\textbf{U}{{\varvec{J}}}$.

As an application of the above, we have the following connection between conjugations and hyperinvariant subspaces.

### Proposition 8.5

Let $\mu \in M_{+}(\mathbb {T})$. If $\mathcal {K}\subseteq \mathscr {L}^2(\mu , \mathcal {H})$ is an invariant subspace for every $\mathfrak {C}\in \mathscr {C}_s({{\varvec{M}}}_\xi )$, then $\mathcal {K}$ is hyperinvariant for ${{\varvec{M}}}_\xi$.

### Proof

For any fixed conjugation *J* on $\mathcal {H}$, (5.4) says that ${{\varvec{J}}}\in \mathscr {C}_s({{\varvec{M}}}_\xi )$ and thus $\mathcal {K}$ is invariant for ${{\varvec{J}}}$. By Theorem 8.4, $\mathcal {K}$ is also invariant for all of the conjugations ${{\varvec{M}}}_u{{\varvec{J}}}$, where $u \in L^\infty (\mu )$ is unimodular $\mu$-almost everywhere. Therefore, $\mathcal {K}$ is invariant for every ${{\varvec{M}}}_u={{\varvec{M}}}_u{{\varvec{J}}}{{\varvec{J}}}$, where $u\in L^\infty (\mu )$ is unimodular. From here, one can argue that $\mathcal {K}$ is also invariant for ${{\varvec{M}}}_{\chi _\Omega }$ for any Borel set $\Omega \subseteq \sigma (U)$ (indeed let $u = 1$ on $\Omega$ and $-1$ on $\mathbb {T}\setminus \Omega$ and note that $\chi _{\Omega } = (1 + u)/2$) and, consequently, for any ${{\varvec{M}}}_v$ where $v\in L^\infty (\mu )$.

Now fix any unitary $U_0$ on $\mathcal {H}$ and let ${\textbf{U}}_0 \equiv U_0 \in \mathscr {L}^{\infty }(\mu , \mathcal {B(\mathcal {H})})$ denote the operator-valued constant function. By our discussion in the introduction (see also Proposition 2.5), $U_0$ is $J_0$–symmetric for some conjugation $J_0$. Let ${{\varvec{J}}}_0$ be the conjugation on $\mathscr {L}(\mu ,\mathcal {H})$ given by $({{\varvec{J}}}_0{{\varvec{f}}})(\xi )=J_0({{\varvec{f}}}(\xi ))$. Therefore, $\mathcal {K}$ is invariant for ${{\varvec{J}}}_0$ and for $\textbf{U}_0{{\varvec{J}}}_0$ and thus for ${\textbf{U}}_0$. Similarly, $\mathcal {K}$ is invariant for $\textbf{U}_0^*$. It follows that $\mathcal {K}$ is invariant for the von Neumann algebra containing all constant unitary valued functions in $\mathcal {B}(\mathcal {H})$. Finally, $\mathcal {K}$ is invariant for the von Neumann algebra generated by $\{{{\varvec{M}}}_{v}: v \in L^{\infty }(\mu )\}$ and the constant operator-valued functions from $\mathscr {L}^{\infty }(\mu , \mathcal {B}(\mathcal {H}))$. From here, one can fashion an argument that $\mathcal {K}$ is invariant for every element of $\mathscr {L}^\infty (\mu , \mathcal {B}(\mathcal {H}))$. Since $\mathscr {L}^\infty (\mu , \mathcal {B}(\mathcal {H}))$ forms the class of operators ${{\varvec{M}}}_{\Phi }$, $\Phi \in \mathscr {L}^\infty (\mu , \mathcal {B}(\mathcal {H}))$, that commute with ${{\varvec{M}}}_{\xi }$, $\mathcal {K}$ is hyperinvariant for ${{\varvec{M}}}_\xi$.

From here, we can state our main connection between conjugations and hyperinvariant subspaces.

### Theorem 8.6

Let *U* be a unitary operator on a separable Hilbert space $\mathcal {H}$. If $\mathcal {M}\subseteq \mathcal {H}$ is an invariant subspace for every $C\in \mathscr {C}_s(U)$, then $\mathcal {M}$ is hyperinvariant for *U*.

### Proof

The notation from Theorem 8.2 proves that the invariance of $\mathcal {M}$ for all $C \in \mathscr {C}_{s}(U)$ implies that $\mathcal {I}\mathcal {M}=\bigoplus \mathcal {K}_i$, where the subspace $\mathcal {K}_i$ is invariant for all all ${{\varvec{M}}}_{\textbf{U}_i}{{\varvec{J}}}_i$, where $\textbf{U}_i\in \mathscr {L}^\infty (\mu _i, \mathcal {H}_i)$ is unitary valued $\mu$-almost everywhere. Now apply Proposition 8.5 to see that $\mathcal {K}_i$ is hyperinvariant for each ${{\varvec{M}}}_{\xi }^{(i)}$. Finally, using the description of the commutant of ${{\varvec{M}}}_{\xi }$ [7, p. 307, Theorem IX.10.20], we obtain that $\mathcal {I}\mathcal {M}$ is hyperinvariant for ${{\varvec{M}}}_{\xi }$. This shows that $\mathcal {M}$ is a hyperinvariant subspace for *U*.

The simple example bellow illustrates why requirement in Theorem 8.6 that $\mathcal {M}$ is invariant for *every* $C\in \mathscr {C}_s(U)$ is an important one.

### Example 8.7

Consider the diagonal unitary operator $U\in \mathcal {B}(\mathbb {C}^2)$ defined by $U=\text {diag}[\lambda ,\lambda ]$ (where $\lambda \not = 0$) with respect to the standard basis $\{\textbf{e}_1,\textbf{e}_2\}$ for $\mathbb {C}^2$. The only hyperinvariant subspaces for *U* are the trivial ones $\{\textbf{0}\}$ and $\mathbb {C}^2$ (since any $2 \times 2$ matrix commutes with *U*). Define two conjugations $C_1$, $C_2$ on $\mathbb {C}^2$ by

$$\begin{aligned} C_1(\textbf{e}_1)= \textbf{e}_1, \; C_1(\textbf{e}_2)= \textbf{e}_2 \quad \text{ and } \quad C_2(\textbf{e}_1)=\textbf{e}_2, \; C_2(\textbf{e}_2) = \textbf{e}_1 \end{aligned}$$

(and of course extend antilinearity to all of $\mathbb {C}^2$). One can check that $C_1,C_2\in \mathscr {C}_s(U)$. Note that each $C_i$ separately has more invariant subspaces than the trivial ones $\{\textbf{0}\}$ and $\mathbb {C}^2$, but only the trivial subspaces are simultaneously invariant for both of them.

Our summary theorem connecting hyperinvariant subspaces and conjugations is the following.

### Theorem 8.8

Let *U* be a unitary operator on a separable Hilbert space $\mathcal {H}$ with spectral measure $E(\cdot )$. For a subspace $\mathcal {M} \subseteq \mathcal {H}$, the following are equivalent.

- $$\mathcal {M}$$ is hyperinvariant;
- $\mathcal {M} = E(\Omega ) \mathcal {H}$ for some Borel set $\Omega \subseteq \sigma (U)$;
- $$\mathcal {M} = \mathcal {H}_{\mu }$$ for some $$\mu \in M_{+}(\mathbb {T})$$;
- $$C \mathcal {M} \subseteq \mathcal {M}$$ for every $$C \in \mathscr {C}_{s}(U)$$.

### Proof

$(a) \Longrightarrow (b)$ is Theorem 3.7. $(b) \Longrightarrow (c)$ is Theorem 3.8. $(c) \Longrightarrow (d)$ is Theorem 3.2. $(d) \Longrightarrow (a)$ is Theorem 8.6.

### Remark 8.9

One can use Theorem 8.2 to give an alternate proof of Theorem 4.2 (the description of $\mathscr {C}_{s}(U)$ when *U* is an $n \times n$ unitary matrix). The same is true for Examples 4.4 and 4.6.
